# Functional diversity positively affects prey suppression by invertebrate predators: a meta‐analysis

**DOI:** 10.1002/ecy.2378

**Published:** 2018-07-05

**Authors:** Arran Greenop, Ben A. Woodcock, Andy Wilby, Samantha M. Cook, Richard F. Pywell

**Affiliations:** ^1^ NERC Centre for Ecology & Hydrology Maclean Building, Crowmarsh Gifford Wallingford Oxfordshire OX10 8BB UK; ^2^ Lancaster Environment Centre Lancaster University Library Avenue Lancaster LA1 4YQ UK; ^3^ Biointeractions and Crop Protection Department Rothamsted Research Harpenden Herts AL5 2JQ UK

**Keywords:** agricultural ecosystems, biodiversity and ecosystem functioning, conservation biological control, ecosystem services, functional diversity, natural enemies, phylogenetic diversity, predator‐prey interactions, species richness, traits

## Abstract

The use of pesticides within agricultural ecosystems has led to wide concern regarding negative effects on the environment. One possible alternative is the use of predators of pest species that naturally occur within agricultural ecosystems. However, the mechanistic basis for how species can be manipulated in order to maximize pest control remains unclear. We carried out a meta‐analysis of 51 studies that manipulated predator species richness in reference to suppression of herbivore prey to determine which components of predator diversity affect pest control. Overall, functional diversity (FD) based on predator's habitat domain, diet breadth and hunting strategy was ranked as the most important variable. Our analysis showed that increases in FD in polycultures led to greater prey suppression compared to both the mean of the component predator species, and the most effective predator species, in monocultures. Further analysis of individual traits indicated these effects are likely to be driven by broad niche differentiation and greater resource exploitation in functionally diverse predator communities. A decoupled measure of phylogenetic diversity, whereby the overlap in variation with FD was removed, was not found to be an important driver of prey suppression. Our results suggest that increasing FD in predatory invertebrates will help maximize pest control ecosystem services in agricultural ecosystems, with the potential to increase suppression above that of the most effective predator species.

## Introduction

The predicted growth of global populations will lead to an ever‐increasing demand for agricultural systems to deliver greater food production (25–75% increase in food by 2050; Hunter et al. 2017). Whilst this goal may be achieved through conventional forms of agricultural intensification, there are likely limitations to the extent to which chemical insecticides can be relied upon without facing a myriad of risks. These range from the likelihood of pesticide resistance in pest species (Nauen and Denholm 2005, Bass et al. 2014), the revocation of active ingredients (NFU, 2014), damaging effects on non‐target organisms (Easton and Goulson 2013, Hallmann et al. 2014, Woodcock et al. 2016, 2017), as well as diffuse pollution impacting on human and environmental health in general (Wilson and Tisdell 2001, Horrigan et al. 2002). An increased reliance on conservation biological control, where predators or parasitoids (here, referred to collectively as predators) of pest species are encouraged within agricultural ecosystems has the potential to address some of these issues (Begg et al. 2017). Fundamental to integrating conservation biological control into agricultural practices is understanding which components of invertebrate biodiversity need to be managed to maximize pest suppression.

A number of meta‐analyses (Bianchi et al. 2006, Letourneau et al. 2009, Griffin et al. 2013) have demonstrated that higher predator richness can increase prey suppression (reduction in herbivores by predators), however, species richness provides little elucidation as to the underlying mechanisms driving this trend. An important characteristic of multi‐predator systems is the presence of significant variation in the response of prey suppression to increasing predator species richness; a consequence of the range of complex interactions between predators, and predators and prey (Ives et al. 2004, Casula et al. 2006, Schmitz 2007). For example, intraguild interactions can be positive (functional facilitation), whereby predators facilitate the capture of prey by other predator species (Losey and Denno 1998). Niche complementarity is another interaction that can lead to overyielding of prey suppression by diverse assemblages, where individual predators may feed on different life stages of a prey species (Wilby et al. 2005). However, negative interactions also occur between predators reducing prey suppression in diverse assemblages. One of the most commonly encountered of these is intraguild predation, whereby a top predator consumes not only the prey but also the intermediate predators (Rosenheim et al. 2004a, Finke and Denno 2005). Interference competition can also occur whereby one predator species reduces prey capture by the other due to negative behavioral interactions (Lang 2003). Given the complexity of these interactions, the net effect of predator species diversity is often difficult to predict.

Defining morphological or behavioral characteristics of individual species that potentially impact on prey suppression, often referred to as functional effect traits, provides an opportunity to elucidate the mechanistic link between predator biodiversity and the delivery of this ecosystem service (Wood et al. 2015). For example, Schmitz (2007) suggested that traits related to habitat domain (the spatial location of where the natural enemy feeds, e.g., ground or upper canopy of vegetation) and hunting method (how they catch prey, e.g., sit & wait) were important in understanding how predator interactions affected prey suppression. Similarly, size differences between predators and prey can also influence intraguild interactions and play an important role in predicting consumption rates (Rosenheim et al. 2004b, Brose et al. 2008, Ball et al. 2015). While these assumptions have been supported in part by several studies (Woodcock and Heard 2011, Miller et al. 2014, Northfield et al. 2014, Michalko and Pekár 2016) the direct implications of functional diversity (FD) between species on their capacity to deliver pest control remains poorly understood.

An understanding of how predator diversity and traits influence pest suppression has been identified by several reviews as being crucial to the implementation of sustainable pest management in agricultural ecosystems (Bianchi et al. 2010, Wood et al. 2015, Jonsson et al. 2017, Perović et al. 2017). This information is a required step in bridging the gap between experimental small‐scale mesocosm (cage) studies and generalizable rules that can be used by practitioners in field‐scale management strategies, and a detailed meta‐analysis directly addressing this question has yet to be undertaken (Woodcock et al. 2013).

Here we address this knowledge gap by undertaking a meta‐analysis to identify how dissimilarity in key functional effects traits of invertebrate predators can influence interactions between predators and their prey to affect pest suppression. The meta‐analysis was undertaken using 51 studies (214 data points) comprising a total of 73 predator species attacking 35 species of arthropod prey. We assess how both FD based on an a priori selection of traits, and phylogenetic diversity (PD) based on evolutionary history are linked to prey suppression (Cadotte et al. 2013). We use the meta‐analysis to test the general prediction that increased predator species richness leads to greater prey suppression (prediction 1) (e.g., Letourneau et al. 2009, Griffin et al. 2013, Katano et al. 2015). We also test the following predictions related to explaining diversity effects; increased FD of key effects traits explains patterns in prey suppression in polycultures due to increased niche complementarity between predator species (prediction 2); PD has a smaller effect on prey suppression than FD as it accounts for broad differences in evolutionary history, compared to FD which is based on an a priori selection of traits (prediction 3); and finally related to body size differences between predators, and predators and prey we predict that, increased body size ratio between predators and prey will positively affect prey suppression, whilst greater size differences between predators will negatively affect prey suppression due to increased intraguild predation (prediction 4) (Lucas et al. 1998, Rosenheim et al. 2004b, Brose 2010, Ball et al. 2015).

## Materials and Methods

### Study selection and data

We carried out a systematic literature search of studies testing the impact of factorial combinations of increasing predator or parasitoid species richness on prey suppression. These experiments were all undertaken in mesocosms, representing an experimental arena within which population changes of the prey species could be monitored. Literature searches were carried out between November 2016 – January 2017 using *ISI Web of Science* (search terms included in Appendix S1 in Supporting Information) and reference lists published in the following studies: Sih et al. (1998), Straub et al. (2008), Letourneau et al. (2009), Griffin et al. (2013), Katano et al. (2015). In addition, unpublished sources (Asiry 2011, Fennel 2013) of literature were included and additional studies identified by E. Roubinet (*Personal communication*).

Studies were selected based on their fulfilment of the following criteria: (1) the study system was of terrestrial arthropods, (2) predator species richness was manipulated in reference to the suppression of arthropod prey species, (3) the study considered two or more predator species, (4) all predators of prey were included in monoculture (species A or species B) and polyculture (species A + B) treatments, (5) the study contained a quantifiable measure of prey suppression, (6) the study included mean, standard deviations and the number of replicates for each treatment. Typically, individual published studies were composed of multiple experiments where factors other than predator species richness were manipulated. These factors included prey species richness, habitat complexity, temperature/environmental conditions, predator life stage, predator density as well as methodological factors such as the use of additive and substitutive experimental designs; of which factors could potentially impact the nature of multi‐predator trophic interactions and the observed outcome on prey suppression (Finke and Denno 2002, Wilby and Orwin 2013, Ajvad et al. 2014, Drieu and Rusch 2017). These experiments were therefore treated as separate data points. For studies investigating responses of multiple instars of the same predator species, only the life stages that provided the maximum and minimum prey suppression were included. This was done to avoid potential pseudo‐replication due to strong functional similarity between successive larval instars while providing an indication of the full range of potential emergent impacts on prey suppression by that species (Cisneros and Rosenheim 1997).

### Quantification of herbivore suppression effect sizes

Where possible, we extracted data on the impact of predator diversity on prey suppression directly from published studies, either from presented data or using WebPlotDigitizer 3.11 (Rohatgi 2012) to extract information from graphs. Where the required information was not available, the raw data was requested directly from the corresponding author. A total of 51 studies constituting 214 data points were included in analyses (see Appendix S2 for literature included). As prey suppression was measured in several different ways, we used the standardized mean difference corrected for small sample sizes as our test statistic (Hedges 1981, Hedges and Olkin 1985). We also calculated the corresponding sampling variance for each experiment (Hedges 1981, Hedges and Olkin 1985). Following Cardinale et al. (2006) and Griffin et al. (2013), we calculated two test statistics for each experimental data point. The first is SMD_mean_, which is the standardised mean difference between the mean ($\overset{¯}{x}$) effect of the predator polyculture (*p*) on prey suppression compared to the mean effect of the component predator species in monocultures (*m*) calculated as:

$$\text{SMD}\;=\;\frac{\bar{x_{p}}-\bar{x_{m}}}{s}J,$$

where *s* is the pooled standard deviation calculated as:

$$s\;=\;\frac{\sqrt{\left(n_{p}-1\right){\text{SD}}_{p}^{2}\;+\;\left(n_{m}-1\right){\text{SD}}_{m}^{2}}}{n_{p}\;+\;n_{m}-2}$$

and *J* a correction factor applied for small sample sizes:

$$J\;=\;\frac{3}{4(n_{p}\;+\;n_{m})-1}$$

The variance (*v*) for each experiment was calculated as:

$$V\;=\;\frac{n_{p}\;+\;n_{m}}{n_{p}n_{m}}\;+\;\frac{{\text{SMD}}^{2}}{2(n_{p}\;+\;n_{m})}$$

The second metric, SMD_max_, is the standardized mean difference between the mean effect of the polyculture on prey suppression compared to the most effective predator species in a monoculture (*m*_*x*_), where *m*_*x*_ replaces *m* in the above equations. Where the measure of prey suppression was negative (e.g., aphid population size decreased due to greater predation) then the sign of the mean was reflected (multiplied by minus 1) so that the measure could be more intuitively interpreted as a positive effect of increased prey suppression in polycultures (Griffin et al. 2013). All effect sizes and sampling variances were calculated in RStudio using the *metafor* package (Viechtbauer 2010, R Core Team, 2016).

### Species richness

Variables were included for predator species richness and prey species richness, as a meta‐analysis by Katano et al. (2015) demonstrated variation in herbivore suppression between different richness levels. Both variables were included as categorical due to a strong skew towards lower richness levels (prey richness = 1 [*n* = 177] and prey richness >1 [*n* = 37]; predator richness = 2 [*n* = 152] and predator richness >2 [*n* = 62]).

### Effects traits describing functional diversity

For each of the predator species we collected information on ‘effects traits’ which represent physical or behavioral characteristics that would have a direct impact on prey suppression. Due to the taxonomic breadth of predator species we included effects traits based on: hunting strategy, defined as the method used by the predator species to capture prey; habitat domain, defined as the part of the experimental area where the predator predominantly hunts; and diet breadth, describing whether the predators were generalists or specialists. The trait categories, definitions and species within these groups are shown in Appendix S3: Table S1, S2. Where possible trait classifications were obtained directly from the study included in the meta‐analysis. Where this was not possible information on species ecology was determined from a search of primary and grey literature, as well as the use of expert opinion. These traits were selected as previous research suggests they play an important role in predator‐predator interactions and the resultant effect on herbivore suppression (Losey and Denno 1998, Schmitz 2007, Straub et al. 2008, Woodcock and Heard 2011, Ball et al. 2015). A Gower dissimilarity matrix (Gower 1971) was calculated using these effects traits. The square root of the Gower dissimilarity matrix was then subjected to principle coordinate analysis and used to calculate mean pairwise dissimilarity between the predator species within each experiment as an index of functional diversity (FD) (see Functional and phylogenetic diversity measures for a description). Functional dissimilarity pairwise matrices were calculated using the *decouple* function supplied in De Bello et al. (2017).

### Phylogentic diversity

Whilst the functional effects traits were selected due to their direct importance in predicting prey suppression based on previous research, these do not describe the full functional identity of individual species. This functional identity would be defined by both response traits as well as potentially undefined effects traits linked to pest control delivery. These between species differences in combined functional characteristics can be explained by phylogenetic history, with the assumption that a common evolutionary origin will explain a large component of the functional similarity in traits that characterize predator species (Cadotte et al. 2013). We used the Linnaean taxonomic classification (phylum, class, order, family, genus) for the predator species to construct a surrogate phylogenetic tree in the *ape* package in RStudio (Paradis et al. 2004). From this tree, a matrix of phylogenetic dissimilarity was calculated from the square root branch lengths between the tips of the tree for each species. The overlap in variation between the functional dissimilarity and phylogenetic dissimilarity between each species was then decoupled using the *decouple* function described in De Bello et al. (2017). This was carried out to ensure that the two measures for each species were explaining unique components of predator diversity. This was then used to derive a decoupled phylogenetic dissimilarity matrix between predator species. The functional diversity metric incorporates diversity linked to both individual traits and an inherent component resulting from phylogenetic links between species (referred to as FDist in De Bello et al. 2017). As such this is typical of other existing functional diversity metrics (for example Rao's quadratic entropy (De Bello et al. 2017)). However, the decoupled phylogenetic diversity metric represents the residual phylogenetic variation not accounted for through the functional traits (referred to as dcPDist in De Bello et al. 2017). This decoupled measure of phylogenetic diversity was included as it allowed us to identify if other unmeasured traits captured by phylogenetic diversity were important in prey suppression.

### Functional and phylogenetic diversity measures

From each functional and phylogenetic dissimilarity matrix, we calculated the mean pairwise dissimilarity between species in each experiment using the *melodic* function supplied in De Bello et al. (2016);

$$\text{Mean pairwise dissimilarity}\;=\;\frac{1}{\sum_{i>j}^{N}p_{i}p_{j}}\sum_{i>j}^{N}p_{i}p_{j}d_{\mathrm{ij}},$$

where *N* is the number of species in a community, *dij* is the dissimilarity between each pair of different species *i* and *j*, respectively, *pi* and *pj* are the relative abundances of species *i* and *j*, respectively, divided by the total of all species abundances in a community. We used an unweighted index based on presence/absence (where *p*_*i*_ **= 1/*N*) as predator numbers were equal in the majority of experiments included in the meta‐analysis. Mean pairwise dissimilarity was selected for all the phylogenetic and functional diversity measures (see Table 1) as it has been found to be relatively insensitive to species richness where richness levels are low (De Bello et al. 2016).

**Table 1:** Species variables included in analysis

Variable	Measure	Description
Functional diversity (FD)	Continuous	Mean pairwise functional dissimilarity between species in each experiment based on the traits included in Appendix S3 (excluding body size)
Hunting strategy	Continuous	Mean pairwise dissimilarity between species in each experiment based on hunting stategy (sit and wait, ambush and pursue or active)
Habitat domain	Continuous	Mean pairwise dissimilarity between species in each experiment based on habitat (ground/base of plant, foliar or broad)
Diet breadth	Continuous	Mean pairwise dissimilarity between species in each experiment based on diet breadth (specialist or generalist)
Phylogenetic diversity (PD)	Continuous	Mean pairwise phylogenetic dissimilarity between species based on Linnaean taxonomic classification decoupled from the functional traits
ratio_large_	Continuous	Body size ratio between the largest predator species and the prey species (largest predator body size/prey body size). Sqrt transformed. Excluded from analysis
ratio_small_	Continuous	Body size ratio between the smallest predator species in the polyculture and the prey species (smallest predator body size/prey body size). Sqrt transformed
Size difference	Continuous	Mean pairwise difference in body size (length in mm) between predator species in each experiment
Prey size (mm)	Continuous	Body length of the prey. Where multiple prey were included in a treatment the mean of their body sizes was used. Log transformed
Predator species richness	Factor (2 or >2)	Two level factor categorising polyculture treatments on whether they contained two predators or more than two predators (max predator species richness = 4)
Prey species richness	Factor (1 or >1)	Two level factor categorising whether one or more than one prey species was present in the study (max prey species richness = 4)

### Body size

Body size has been shown to influence predator‐predator interactions where large body sized generalist predators may consume smaller predators as well as prey (Lucas et al. 1998, Rosenheim et al. 2004b). Additionally, body size ratios between predators and prey have been shown to affect consumption rates (Lucas et al. 1998, Rosenheim et al. 2004b, Brose 2010, Ball et al. 2015). We defined a mean body size (body length in mm) for each predator species (Appendix S3). Where different life stages of single predator species were used in experiments, this was accounted for with life‐stage specific mean body size. We also included a mean body size for each of the prey species. From these measures of body size, we calculated the mean size difference in predator body sizes, and the ratio between the smallest predator and prey body size (Table 1). We did not include the individual sizes of smallest and largest predators as covariates as these were both highly inter‐correlated with either predator‐predator size differences or predator‐prey body size ratios (see Appendix S4: Table S1). Similarly, a high level of collinearity was also found between the prey and the largest predator body size ratio (ratio_large_), and prey and the smallest predator size ratio (ratio_small_) variables. The highest ranked model sets including ratio_small_ had lower AIC_c_ scores than the highest ranked ratio_large_ models; therefore only ratio_small_ was included in final analysis (Appendix S4: Table S2–S5).

### Experimental factor moderator variables

In addition to factors associated with predator and prey species richness and traits, a number of experimental factors were also included in analysis that have previously been shown to influence prey suppression. These included: experimental arena volume (cm^3^; log transformed to improve linearity), duration of study following predator addition (hours) and study setting (field, or greenhouse/lab). Additionally, a factor was included to test between study designs (additive or substitutive) as this has been shown to lead to different conclusions about prey suppression depending on the design used (Schmitz 2007, Byrnes and Stachowicz 2009). Additive studies increase the number of predators in the polyculture based on the sum of the component predators in monocultures, whereas substitutive designs maintain the same number of predators in polycultures and monocultures.

### Statistical analysis

Intercept only random effects models were used for both SMD_mean_ and SMD_max_ to determine whether there was an overall effect of greater prey suppression in polycultures. Models included study identity as a random factor to account for the fact that multiple points came from single studies. The restricted maximum likelihood was used (REML) to estimate between study variance. The meta‐analysis was unweighted as weighting by inverse variance has been shown to result in bias against small sample sizes (Hedges and Olkin 1985, Letourneau et al. 2009). All meta‐analyses were undertaken using the rma.mv function in the package *metafor* (Viechtbauer 2010, RStudio, 2015). Wald‐type 95% confidence intervals are given. Assessments of publication bias in response to an underrepresentation of non‐significant results were undertaken using funnel plots (Koricheve et al. 2013). Some evidence of publication bias was found whereby studies with lower precision were more likely to detect negative effects for SMD_max_ (See Appendix S5). However, as this result was not detected for SMD_mean_, this is likely caused by the calculation of the SMD_max_ metric (see Schmid et al. 2008).

We used a meta‐regression with a maximal model including FD, PD, ratio_small_, predator size difference, prey size, prey richness and predator richness to quantify how emergent effects on prey suppression were effected by aspects of invertebrate community structure (Table 1). The response variables were the two metrics SMD_mean_ and SMD_max_. An information theoretic approach was used to identify the best set of candidate models from the full model and we then used multi‐model averaging to obtain parameter estimates (Burnham and Anderson 2004). Maximum‐likelihood was used to allow model comparison with a study subject identifier included as a random effect. All possible model combinations of the variables included in the full model were run. Models that had ΔAIC_c_ values of <2 were then used to rank variable importance and obtain model averaged parameter estimates based on AIC_c_ relative importance weights (Burnham and Anderson 2004). Variables were transformed where required to improve linearity (Table 1). All model averaging was carried out in the *glmulti* package in RStudio (Calcagno and De Mazancourt 2010).

Whilst the FD metric allowed for comparisons to be made to phylogenetic diversity, the inclusion of a number of different traits meant it was difficult to discern which aspects of FD were driving any potential trends. To account for this, we analysed differentiation within each trait using mixed models comparing all possible model combinations based on AIC_c_ values. Full models started with diet breadth, hunting strategy and habitat domain included as fixed effects with the study subject identifier as a random effect. Models that had ΔAIC_c_ of <2 were then ranked to obtain model‐averaged parameter estimates based on AIC_c_ relative importance weights (Burnham and Anderson 2004). Models were also run including just FD, so that a comparison of AIC_c_ values of the individual traits with the composite metric of functional diversity could be made.

We also individually tested whether the experimental moderator variables had a significant effect on the two SMD metrics using mixed effects models, again using REML with a study subject identifier included as a random factor. We did not include experimental variables in model averaging as the focus of this analysis was to identify the importance of factors related to predator and prey community structure on prey suppression, not experimental design. Variables were tested individually as information was absent from several studies for some of the experimental explanatory variables.

## Results

### General effects across studies

Overall trends showed greater prey suppression in predator polycultures compared to the mean effect of the component species in a monoculture (SMD_mean_), as the average effect size for SMD_mean_ was significantly greater than zero (SMD_mean_ = 0.444; 95% CI [0.265, 0.623]; *Z* = 4.858, *P* = <0.001). However, SMD_max_ (suppression of herbivores in the polyculture compared to the most effective predator) was not found to differ significantly from zero with a mean effect size of −0.109 (95% CI [−0.308, 0.090], *Z* = −1.078, *P* = 0.281). This shows that increased predator richness in polycultures did not result in significantly greater levels of prey suppression than the most effective predator in a monoculture.

### Predator and prey variables

#### SMD_mean_

**Figure 1:**
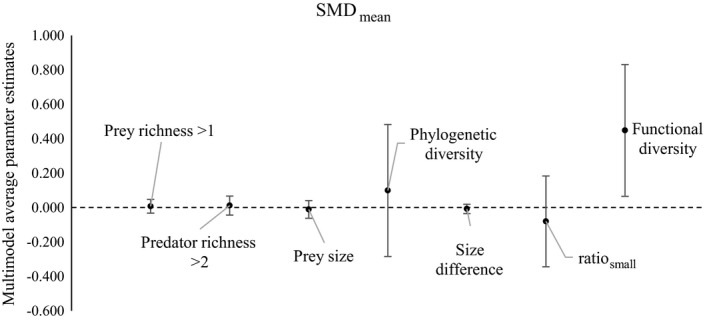
Multimodel average parameter estimates for SMD_mean_ (predator polyculture compared to the mean of the component predator species in monocultures); lines indicate ±95% confidence intervals. Predator richness and prey richness are factors and show the difference between the reference level (reference level for predator richness = 2 species and prey richness = 1 species).

Functional diversity was ranked as the most important variable based on relative model weights of the 2AIC_c_ subset, and was the only parameter included in the top ranked model (Table 2; Fig. 1) (See Appendix S6 for 2AIC_c_ subset). Functional diversity (parameter estimate = 0.448, 95% CI [0.065, 0.831]) had a positive effect on SMD_mean_. Ratio_small_ (parameter estimate = −0.080, 95% CI [−0.344, 0.184]) was ranked as the second most important variable, however had confidence intervals that overlapped zero, as did the variables prey richness, predator richness, size difference, prey size and decoupled phylogenetic diversity (Table 2; Fig. 1).

**Table 2:** Multimodel average parameter estimates for SMD_mean_ (predator polyculture compared to the mean of the component predator species in monocultures) and SMD_max_ (predator polyculture compared to the most effective predator species in a monoculture)

Metric	Parameter	Estimate	Importance	95% CI lower bound	95% CI upper bound
SMD_mean_	Prey richness >1	0.007	0.062	−0.033	0.047
	Predator richness >2	0.011	0.120	−0.044	0.066
	Prey size	−0.011	0.133	−0.062	0.04
	Phylogenetic diversity	0.099	0.233	−0.284	0.482
	Size difference	−0.008	0.320	−0.035	0.019
	ratio_small_	−0.080	0.336	−0.344	0.184
	**Functional diversity**	0.448	1.000	0.065	0.831
SMD_max_	Phylogenetic diversity	0.038	0.122	−0.147	0.223
	Prey size	−0.032	0.211	−0.149	0.085
	Size difference	−0.005	0.245	−0.026	0.016
	**ratio**_**small**_	−0.282	0.747	−0.754	0.190
	**Predator richness >2**	−0.276	1.000	−0.541	−0.011
	**Functional diversity**	0.461	1.000	0.049	0.873

*Notes*

Prey richness and predator richness estimate is the difference between the reference level (predator richness = 2 species; prey richness = 1). Parameters in bold indicate that the variable was included in the highest ranked model.

Where the individual traits were analyzed separately, diet breadth was the only variable included in the top ranked model (See Appendix S7: Table S1). Differentiation within diet breadth (parameter estimate = 0.371, 95% CI [0.096, 0.646]) was found to have a positive effect on SMD_mean_. Hunting strategy was also included in the 2AIC_c_ subset, however had 95% confidence intervals that overlapped zero (hunting parameter estimate = 0.023, 95% CI [−0.098, 0.144]). The FD only model showed a positive effect of FD (parameter estimate = 0.453, 95% CI [0.072, 0.831]). When compared to the diet breadth only model, the FD model had a higher AIC_c_ value (Diet breadth only model AIC_c_ = 443.960; Functional diversity model AICc = 445.671). Suggesting that the beneficial effects of FD on SMD_mean_ in the main predator and prey model may have largely been driven by differentiation in diet breadth.

#### SMD_max_

**Figure 2:**
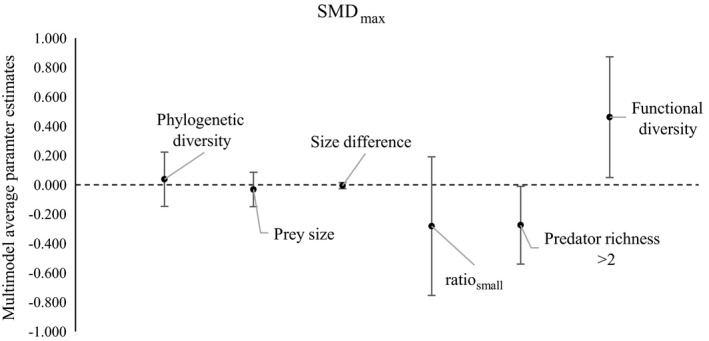
Multimodel average parameter estimates for SMD_max_ (predator polyculture compared to the most effective predator species in a monoculture); lines indicate ±95% confidence intervals. Predator richness is the difference between the reference level (predator richness = 2 species).

Functional diversity, predator richness and ratio_small_ were all included in the top ranked model for SMD_max_ (Appendix S6). Functional diversity (parameter estimate = 0.461, 95% CI [0.049, 0.873]) was again found to have a positive effect, whereas both predator richness of >2 species (parameter estimate = −0.276, 95% CI [−0.541, −0.011]) and ratio_small_ (parameter estimate = −0.282, 95% CI [−0.754, 0.190]) had a negative effect on SMD_max_ (although the 95% CI for ratio_small_ overlapped zero). Variables also included in the top ranked models were prey size and size difference between predators, however, these were only included in models in combination with functional diversity and had confidence intervals that overlapped zero (Table 2; Fig. 2). Decoupled phylogenetic diversity was included in one model in the 2AIC_c_ subset, however it too had confidence intervals that overlapped zero (Table 2; Fig. 2).

Where the traits were analyzed separately, a null model was included in the 2AIC_c_ subset (Appendix S7: Table S4). This indicated that none of the individual traits explained a greater amount of the variation than a model without any factors included. In comparison to the trait model, the FD model showed a clear positive effect of FD (parameter estimate = 0.458, 95% CI [0.049, 0.867]) on SMD_max,_ and had a lower AIC_c_ by a value of <2 compared to the null model (Appendix S7). This indicates that the positive effect of FD on SMD_max_ is likely dependent on a composite measure of diversity including all three traits.

### Experimental factors

**Figure 3:**
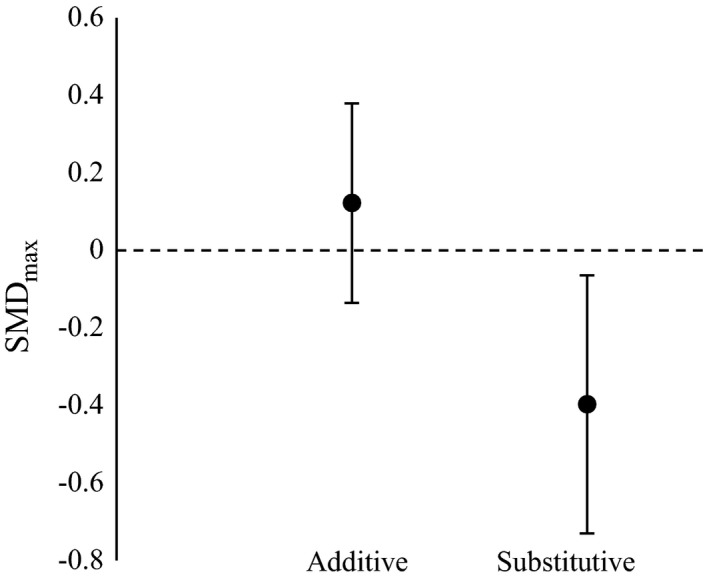
SMD_max_ (predator polyculture compared to the most effective predator species in a monoculture) for additive (*n* = 99) and substitutive (*n* = 115) designs; lines indicate ±95% confidence intervals.

Of the experimental variables tested, study design (additive or substitutive) was found to have a significant effect on SMD_max_ metric (Table 3). Compared to additive designs, substitutive designs were found to have a significantly lower mean effect size (whilst the mean for additive designs was positive, the 95% CI still overlapped zero) (Table 3; Fig. 3). As this is indicative of a potential density effect, where positive diversity effects in polycultures could be a product of predator densities, we re‐analyzed the predator and prey variables for SMD_max_ only including studies that accounted for density. This had no qualitative effect on our results (See Appendix S8). None of the other experimental variables included had a significant effect on SMD_mean_ or SMD_max_, suggesting that the results were not artefacts of differences in spatio‐temporal scale or the study setting (Table 3).

**Table 3:** Tests for experimental moderator variables

Metric	Factor	*n*	Estimate	95% CI lower bound	95% CI upper bound	QM	df	*P*‐value
SMD_mean_	Log cage volume (cm^3^)	186	0.049	−0.018	0.116	2.084	1	0.149
	Duration of study (hours)	209	−0.0002	−0.001	0.0002	0.892	1	0.345
	Design					3.188	1	0.074
	Additive (reference)	99	0.569	0.341	0.797			
	Substitutive	115	−0.277	−0.581	0.027			0.074
	Study setting					0.191	1	0.662
	Field (reference)	89	0.487	0.222	0.752			
	Lab/Greenhouse	125	−0.072	−0.393	0.250			0.662
SMD_max_	Log cage volume (cm^3^)	186	0.037	−0.036	0.109	0.988	1	0.320
	Duration of study (hours)	209	−0.0002	−0.001	0.0003	0.707		0.401
	Design					9.351	1	**0.002**
	Additive (reference)	99	0.122	−0.136	0.379			
	Substitutive	115	−0.519	−0.852	−0.186			**0.002**
	Study setting					0.003	1	0.955
	Field (reference)	89	−0.104	−0.392	0.185			
	Lab/Greenhouse	125	−0.010	−0.353	0.333			0.955

*Notes*

Parameter estimates are shown for continuous variables. Categorical variable estimate is the reference level then the difference between the other levels of the factor. QM statistic is the omnibus test for the factors and Wald *z*‐tests show differences between levels. SMD_mean_ is predator polyculture compared to the mean of the component predator species in monocultures. SMD_max_ is the predator polyculture compared to the most effective predator species in a monoculture. Bold values indicates statistically significant.

## Discussion

When compared to the pest suppression achieved by individual predator species, combining predators in polycultures increased the top‐down control of herbivores. This is consistent with our first prediction that increased predator species richness leads to greater prey suppression. However, this was only the case when considering the average level of prey suppression across all predators (SMD_mean_), with polyculture effects not exceeding those of the most effective predator (SMD_max_). Interestingly, increased species richness above that of simple two predator systems was shown to have a negative effect when polycultures were compared to the most effective predator species. This result is likely an artefact of bias in the calculation of SMD_max_ metric (Schmid et al. 2008, Griffin et al. 2013). Where predator assemblages are species rich they are increasingly likely to include species that affect the extreme ranges of prey suppression. Therefore, whilst sampling effects increase the likelihood that diverse polycultures will include a highly effective predator, when polycultures are compared to the most effective predator in a monoculture, they may be as probable to perform badly due to an increased likelihood of poorly performing predatory species also being present (Schmid et al. 2008). In an agricultural context, this would suggest that management should be targeted towards the most effective predator species rather than increasing overall richness (Straub and Snyder 2006, Straub et al. 2008).

However, the results of our meta‐regression supported our second prediction that greater FD positively affects prey suppression. Further analysis, where we compared the polyculture to the mean of the component species in monocultures, revealed that this was most likely to be driven by differences in diet breadth. Several studies suggest that intraguild predation by generalists on specialist predators can lead to herbivore communities being released from predation (e.g., Rosenheim et al. 1993, Hodge 1999, Snyder and Ives 2001). However, our analysis would suggest that the combination of both generalist and specialist predators in polyculture treatments can lead to greater prey suppression than the mean of the component species. A number of mechanisms are proposed for this; firstly, complementary predation may occur between a generalist predator and specialist parasitoids where the predator prefers feeding on alternate or unparasitized prey, thus minimizing intraguild predation on the parasitoid (Cardinale et al. 2003, Snyder et al. 2004). Secondly, it is possible that spatial resource partitioning commonly occurs between generalist and specialist predators feeding on different parts of the plant (Northfield et al. 2010, Gable et al. 2012). Consequently, our metric of diet breadth may have captured more subtle separation in predator feeding locations between specialist and generalists that were not captured by broader distinction within the habitat domain category. Thirdly, through sampling effects alone, a polyculture containing both specialist and generalist predators may lead to greater prey suppression when compared to the mean of the component species, due to inclusion of the most effective predator. Thus, in our analysis, this may have led to polycultures with increased diversity in the diet breadth category causing greater prey depletion than the mean of the component predator species. Where this occurs positive sampling effects cannot be ruled out. This mechanism is supported by empirical evidence from Straub and Snyder (2006), who found that the inclusion of an aphid specialist within polycultures led to significantly greater aphid depletion than communities without the specialist present. Finally, communities made up of both generalist and specialist predators may provide more stable herbivore control than monocultures of either type of predator alone due to the insurance hypothesis (Snyder et al. 2006).

When we compared polycultures to the most effective predator, none of the single traits (diet breadth, habitat domain and hunting strategy) had a clear effect on prey suppression. Instead, only the composite measure of the functional diversity FD had a positive effect. Functional diversity based on these traits is likely to reflect broad niche partitioning between predators leading to fewer antagonistic interactions, and greater exploitation of available resources (Ives et al. 2004, Finke and Snyder 2008, Northfield et al. 2010, 2017, Gontijo et al. 2015). Previous meta analyses by Cardinale et al. (2006) and Griffin et al. (2013) found that increased predator species richness provided greater prey suppression than the mean of the component species, but not to a greater extent than the most effective predator. The results of our main meta‐analysis are consistent with these studies, however, we have built on this previous research to suggest conditions under which predator polycultures can provide greater prey suppression than the most effective predator, as a result of functional diversity effects mediated through aggregate effects traits. Griffin et al. (2013) used taxonomic distinctness (similar to our measure of phylogenetic diversity) as a proxy for functional diversity and found it had a positive effect on prey suppression in polycultures when compared to the mean of the component species, but not when compared to the most effective predator. In our analysis, when phylogeny was decoupled from aspects of FD it was found to have no clear effect on prey suppression, supporting our third prediction that PD has a smaller effect on prey suppression than FD. One of the reasons that phylogeny was not identified as an important driver of prey suppression may be because only a few effects traits impact on prey suppression in the context of mesocosm studies, and these traits were represented through the FD metric in our analysis. Phylogenetic diversity is often used as a surrogate to represent all functional differences between species, however the variation explained by the key effects traits can be concealed by irrelevant traits also encompassed within the metric, which are a result of divergent evolutionary histories. This has led to contradicting results among different studies. For example, a study by Rusch et al. (2015) found that functional traits selected a priori, based on their link to prey suppression, better predicted aphid pest control compared to a taxonomic approach. Whereas a study by Bell et al. (2008) selected broad ranging functional traits that were incorporated into a single metric and had little effect in predicting the predation rates of a range of invertebrate predators compared to using taxonomy. Therefore, careful consideration of appropriate functional traits would appear imperative to discerning biodiversity and ecosystem functioning relationships where multiple traits are incorporated into a single metric. Furthermore, the relative usefulness of phylogenetic diversity/taxonomic approaches in predicting ecosystem services are also limited by the fact that they do not allow a direct link between traits and a function to be ascertained. This does not preclude the importance of phylogeny between species being of general importance, however in the case of prey suppression where appropriate traits were identified PD did not have a clear effect.

Previous literature suggests that hunting mode and habitat domain play important roles in emergent impacts on prey suppression. However, in the current meta‐analysis neither trait was identified to be individually important. The absence of detected effects of these traits within this meta‐analysis may be due to limitations in the data set. For example, biases in the source data meant that ‘'sit and wait’’ and ‘’mobile‐active’’ predators occurring within the same habitat made up a small proportion (18%) of the studies included in the analysis. This would limit the capacity of the analysis to differentiate between effects of these hunting modes. A further issue may relate to how well broad habitat categorizations capture fine scale differences in predator's habitat use across diverse study systems. It is possible that while the application of hunting domain and habitat domain to predict overyielding is effective, its definition within these categories needs to be defined on a community by community basis. Independent of these issues linked to limitations in the data, our results still suggest that broad niche differentiation through FD leads to overyielding. It is highly likely that this is at least in part a function of complementarity between predators within combinations of habitat domain, hunting mode and/or the diet preferences. This study ultimately provides evidence for the importance of predator functional diversity as a prerequisite for effective pest control across compositionally different predator‐prey systems. However, pulling apart the exact nature of the mechanisms that underpin this will be dependent on new methodological approaches to classification of factors like hunting strategy and habitat domain that allow for making high resolution comparisons between fundamentally different predator‐prey systems. Northfield et al. (2017) present a spatially explicit theory to describe predator interactions across landscapes that is not dependent on temporal or spatial scale. They suggest that where there is complete overlap in spatial resource utilization between predators, antagonistic interactions are likely to decrease the capacity of predators to suppress herbivore prey. Our results, whilst not from a spatially explicit standpoint, also broadly suggest that separate resource utilization by predators will promote positive intraguild interactions across diverse systems.

In contradiction to our fourth prediction, we found an increase in the body size ratio between the smallest predator and prey species had a negative impact on prey suppression in polycultures, although there was large variation within this result. This is surprising as consumption rates and handling times are predicted to be larger and smaller, respectively, where the size difference between a predator and its prey is large (Petchey et al. 2008, Ball et al. 2015). A possible explanation is that as animals with larger body sizes tend to consume prey with a wider range of body sizes (Cohen et al. 1993), top generalist predators may consume smaller predators as well as prey where the difference in energy gain between prey items is large (Heithaus 2001, Lima 2002). However, it could have been expected that the size difference variable between predators would have had a greater effect in our analysis. Size differences between predators may become more important where predators occupy the same habitat and show little specialization in diet breadth. For example, Rusch et al. (2015) found that size differences weakened pest suppression in predatory ground beetles, which not only occur in the same habitat domain but are also generalist predators.

Our meta‐analysis highlights the importance of trait identification when discerning the relationships between biodiversity and ecosystem functioning, i.e., true effects traits like diet breadth, hunting strategy and habitat domain as used in this study that have been shown in quantitative research to play a direct role in the provision of an ecosystem service (Losey and Denno 1998, Schmitz 2007, Straub et al. 2008, Woodcock and Heard 2011, Ball et al. 2015). Understanding how species will respond to environmental perturbation through key response traits and how this will in turn affect functioning through fluctuations in effects traits is important in ascertaining the stability of ecosystem services in a changing environment (Oliver et al. 2015, Jonsson et al. 2017, Perović et al. 2017). Theoretically, where FD is concomitant with redundancy amongst predators and there is little correlation between response and effects traits, this should provide greater stability of pest control ecosystem services (Oliver et al. 2015). This is because systems are more resilient to the loss of individual predators as long as their functions are maintained within the ecosystem (Oliver et al. 2015). However, whilst redundancy should theoretically lead to greater ecosystem service stability, this does not always occur. For example, functional redundancy between parasitoids species was not found to improve the temporal stability of parasitism rates, with food web connectivity appearing more important in stability (Peralta et al. 2014). Consequently, more research is needed to determine the role of FD and functional redundancy in ecosystem service stability.

Of the experimental variables, only study design (additive vs. substitutive) had a significant effect on prey suppression. Prey suppression in polycultures compared to monocultures was lower in substitutive than additive designs. The predominant reason for this could be that higher predator density in additive experimental polycultures may increase prey suppression where predation rates are density dependent and intraspecific interactions between heterospecific predators are neutral or positive (Griffen 2006). Importantly, this also highlights the possibility that increasing predator density within agro‐ecosystems has beneficial effects on pest suppression.

## Conclusion

Our results suggest that maximizing functional diversity in predatory invertebrates within agricultural ecosystem will improve natural pest control. Relatively simple management measures, such as the inclusion of tussock‐forming grasses in buffer strips surrounding crop fields, have been found to increase the FD of ground beetle assemblages on arable farmland (Woodcock et al. 2010). However, it is currently difficult to advocate single management options as other field margin types, such as grass leys, have conversely been found to increase the functional similarity in spider communities (Rusch et al. 2014). It is therefore likely that habitat complexity plays an important role with a diversity of non‐crop habitats needed to promote FD across a wide range of predators (Woodcock et al. 2010, Lavorel et al. 2013, Rusch et al. 2016). However, it is difficult to ascertain the precision with which this can be achieved in practice. Whilst mesocosms are useful for identifying basic species interactions they represent a simplified environment. Real‐world agricultural ecosystems are host to an array of predator and pest species with complex life cycles. Mesocosm studies fail to account for fluctuations in predator numbers/assemblages both spatially and temporally. Therefore, traits related to phenology and dispersal are likely to be relevant in field conditions and would be important to consider in any management practices (Landis et al. 2000). The results of our meta‐analysis fall short of identifying a generalizable rule across all predator interactions that lead to overyielding. However, the findings do highlight the need to quantify how important context is, in terms of predator community assemblage and habitat, in determining which trait combinations promote beneficial effects from functional diversity for pest control ecosystem services. Future studies should aim to identify complimentary sets of traits within different predator communities to determine whether certain trait combinations consistently lead to overyielding, or whether the context dependency of differing predator communities and habitat means that the importance of different trait combinations fluctuates depending on the ecological setting. As we found no clear effects of individual traits, and only our overall metric of FD affected overyielding, our results would suggest that the latter is more likely. However, further research is required in realistic field based studies to determine this.
