# Supplementary material for: Functional diversity positively affects prey suppression by invertebrate predators: a meta‐analysis
Source: Ecology. 2018 Jul 5;99(8):1771–82. doi: 10.1002/ecy.2378 (PMC6099248; doi:10.1002/ecy.2378)
Supplement: Supplementary file 1 [file ECY-99-1771-s001.docx]

**Appendix S1**

Search terms used in web of science:

(predator OR predation OR natural enemy OR parasitoid) AND (richness OR biodiversity OR diversity) AND (pest OR prey OR suppression OR biocontrol OR biological control OR ecosystem function* OR ecosystem process* OR diversity-function) AND (insect* or invertebrate*) AND (experiment OR experimental OR manipulation)

(predator OR predation OR natural enemy OR parasitoid) AND (pest OR prey OR suppression OR biocontrol OR biological control OR ecosystem function* OR ecosystem process* OR diversity-function) AND (insect* or invertebrate*) AND (experiment OR experimental OR manipulation OR cage OR mesocosm)
