# Supplementary material for: Functional diversity positively affects prey suppression by invertebrate predators: a meta‐analysis
Source: Ecology. 2018 Jul 5;99(8):1771–82. doi: 10.1002/ecy.2378 (PMC6099248; doi:10.1002/ecy.2378)
Supplement: Supplementary file 2 [file ECY-99-1771-s002.docx]

**Appendix S2**

Studies included in the meta-analysis.

Acheampong, S. & Stark, J.D. (2004). Can reduced rates of pymetrozine and natural enemies control the cabbage aphid, Brevicoryne brassicae (Homoptera: Aphididae), on broccoli? *International Journal of Pest Management*, 50, 275–279.

Alhmedi, A., Haubruge, E. & Francis, F. (2010). Intraguild interactions and aphid predators: biological efficiency of *Harmonia axyridis* and *Episyrphus balteatus*. *Journal of Applied Entomology*, 134, 34–44.

Ajvad, F.T., Madadi, H. and Gharali, B. (2014). Influence of intraguild predation between Episyrphus balteatus and Hippodamia variegata on their prey. *Arch. Phytopathol. Plant Protection*, 47, 106-112.

Aquilino, K.M., Cardinale, B.J. & Ives, A.R. (2005). Reciprocal effects of host plant and natural enemy diversity on herbivore suppression: an empirical study of a model tritrophic system. *Oikos*, 108, 275–282.

Asiry, K.A.M. (2011). The impacts of cereal-legume intercropping on biological control of cereal aphids within temperate agro-ecosystems. Doctoral Thesis. University of Reading.

Ball, S.L., Woodcock, B.A., Potts, S.G. & Heard, M.S. (2015). Size matters: Body size determines functional responses of ground beetle interactions. *Basic and Applied Ecology*, 16, 621–628.

Bjorkman, C. & Liman, A.-S. (2005). Foraging behaviour influences the outcome of predator-predator interactions. *Ecological Entomology*, 30, 164–169.

Cardinale, B.J., Harvey, C.T., Gross, K. & Ives, A.R. (2003). Biodiversity and biocontrol: emergent impacts of a multi-enemy assemblage on pest suppression and crop yield in an agroecosystem. *Ecology Letters*, 6, 857–865.

Cardinale, B.J., Weis, J.J., Forbes, A.E., Tilmon, K.J. & Ives, A.R. (2006). Biodiversity as both a cause and consequence of resource availability: a study of reciprocal causality in a predator-prey system. *Journal of Animal Ecology*, 75, 497–505.

Chang, G.C. (1996). Comparison of Single Versus Multiple Species of Generalist Predators for Biological Control. *Environmental Entomology*, 25, 207–212.

Cisneros, J. & Rosenheim, J. (1997). Ontogenetic change of prey preference in the generalist predator Zelus renardii and its influence on predator-predator interactions. *Ecological Entomology*, 22, 399–407.

Denno, R.F., Mitter, M.S., Langellotto, G.A., Gratton, C. & Finke, D.L. (2004). Interactions between a hunting spider and a web-builder: consequences of intraguild predation and cannibalism for prey suppression. *Ecological Entomology*, 29, 566–577.

Drieu, R. and Rusch, A. (2016). Conserving species‐rich predator assemblages strengthens natural pest control in a climate warming context. *Agricultural and Forest Entomology*, 19, 52-59.

Dinter, A. (2002). Microcosm studies on intraguild predation between female erigonid spiders and lacewing larvae and influence of single versus multiple predators on cereal aphids. *Journal of Applied Entomology*, 126, 249-257.

Erbilgin, N., Dahlsten, D.L. & Chen, P. (2004). Intraguild interactions between generalist predators and an introduced parasitoid of Glycaspis brimblecombei (Homoptera: Psylloidea). *Biological Control*, 31, 329–337.

Evans, E.W. (1991). Intra versus interspecific interactions of ladybeetles (Coleoptera: Coccinellidae) attacking aphids. *Oecologia*, 87, 401–408.

Fennel, J. (2013). Does host-plant nutrient supply affect the interaction of arthropod predators in a multi-species assemblage? Unpublished dissertation. Lancaster University.

Ferguson, K.I. & Stiling, P. (1996). Non-additive effects of multiple natural enemies on aphid populations. *Oecologia*, 108, 375–379.

Finke, D.L. & Denno, R.F. (2002). Intraguild Predation Diminished in Complex-Structured Vegetation: Implications for Prey Suppression. *Ecology*, 83, 643.

Finke, D.L. & Denno, R.F. (2003). Intra-guild predation relaxes natural enemy impacts on herbivore populations. *Ecological Entomology*, 28, 67–73.

Finke, D.L. & Denno, R.F. (2005). Predator diversity and the functioning of ecosystems: the role of intraguild predation in dampening trophic cascades. *Ecology Letters*, 8, 1299–1306.

Flowers, R.W., Salom, S.M. & Kok, L.T. (2005). Competitive Interactions Among Two Specialist Predators and a Generalist Predator of Hemlock Woolly Adelgid, *Adelges tsugae* (Homoptera: Adelgidae), in the Laboratory. *Environmental Entomology*, 34, 664–675.

Gable, J.T., Crowder, D.W., Northfield, T.D., Steffan, S.A. and Snyder, W.E., (2012). Niche engineering reveals complementary resource use. *Ecology*, 93, 1994-2000.

Gardiner, M.M. & Landis, D.A. (2007). Impact of intraguild predation by adult Harmonia axyridis (Coleoptera: Coccinellidae) on Aphis glycines (Hemiptera: Aphididae) biological control in cage studies. *Biological Control*, 40, 386–395.

Hogg, B.N. and Daane, K.M. (2011). Diversity and invasion within a predator community: impacts on herbivore suppression. *Journal of Applied Ecology*, 48, 453-461.

Hogg, B.N. & Daane, K.M. (2014). The roles of top and intermediate predators in herbivore suppression: contrasting results from the field and laboratory. *Ecological Entomology*, 39, 149–158.

Hogg, B.N. & Daane, K.M. (2015). Cascading effects of cannibalism in a top predator. *Ecological Entomology*, 40, 805–813.

Hogg, B.N., Wang, X.-G., Levy, K., Mills, N.J. & Daane, K.M. (2013). Complementary effects of resident natural enemies on the suppression of the introduced moth Epiphyas postvittana. *Biological Control*, 64, 125–131.

Lester, P.J. & Harmsen, R. (2002). Functional and numerical responses do not always indicate the most effective predator for biological control: an analysis of two predators in a two-prey system. *Journal of Applied Ecology*, 39, 455–468.

Losey, J.E. & Denno, R.F. (1998). Positive Predator-Predator Interactions: Enhanced Predation Rates and Synergistic Suppression of Aphid Populations. *Ecology*, 79, 2143-2152

Lucas, É. & Alomar, O. (2002). Impact of the presence of Dicyphus tamaninii Wagner (Heteroptera: Miridae) on whitefly (Homoptera: Aleyrodidae) predation by Macrolophus caliginosus (Wagner) (Heteroptera: Miridae). *Biological Control*, 25, 123–128.

Mallampalli, N., Castellanos, I. & Barbosa, P. (2002). Evidence for intraguild predation by Podisus maculiventris on a ladybeetle, Coleomegilla maculata: Implications for biological control of Colorado potato beetle, Leptinotarsa decemlineata. *BioControl*, 47, 387-398

Lewins, S.A. (2006). The Influence of Predator Species Richness on Prey Mortality: Implications to Conservation Biological Control. Unpublished Masters Thesis. University of Maryland

Moreno-Ripoll, R., Gabarra, R., Symondson, W.O.C., King, R.A. and Agustí, N. (2014). Do the interactions among natural enemies compromise the biological control of the whitefly Bemisia tabaci?. *Journal of Pest Science*, 87, 133-141.

Northfield, T.D., Snyder, G.B., Ives, A.R. & Snyder, W.E. (2010). Niche saturation reveals resource partitioning among consumers. *Ecology Letters*, 13, 338–348

Obrycki, J.J., Giles, K.L. & Ormond, A.M. (1998). Experimental assessment of interactions between larval *Cole-omegilla maculata* and *Coccinella septempunctata* (Coleoptera : Coccinellidae) in field cages. *Environmental Entomology*, 27, 1280–1288.

Rosenheim, J.A. (2001). Source-Sink Dynamics for a Generalist Insect Predator in Habitats with Strong Higher-Order Predation. *Ecological Monographs*, 71, 93-116

Rosenheim, J.A., Limburg, D.D., Colfer, R.G., Fournier, V., Hsu, C.L., Leonardo, T.E. & Nelson, E.H. (2004a). Herbivore population suppression by an intermediate predator, Phytoseiulus macropilis, is insensitive to the presence of an intraguild predator: an advantage of small body size? *Oecologia*, 140, 577–585.

Rosenheim, J.A., Glik, T.E., Goeriz, R.E. & Rämert, B. (2004b). Linking a predator’s foraging behavior with its effects on herbivore population suppression. *Ecology*, 85, 3362–3372.

Rosenheim, J.A., Wilhoit, L.R. & Armer, C.A. (1993). Influence of intraguild predation among generalist insect predators on the suppression of an herbivore population. *Oecologia*, 96, 439–449.

Roubinet, E., Straub, C., Jonsson, T., Staudacher, K., Traugott, M., Ekbom, B. & Jonsson, M. (2015). Additive effects of predator diversity on pest control caused by few interactions among predator species. *Ecological Entomology*, 40, 362–371.

Sigsgaard, L. (2007). Early season natural control of the brown planthopper, Nilaparvata lugens: the contribution and interaction of two spider species and a predatory bug. *Bulletin of Entomological Research,* 97, 533–544.

Snyder, W.E. & Wise, D.H. (2001). Antipredator behavior of spotted cucumber beetles (Coleoptera : Chrysomelidae) in response to predators that pose varying risks. *Environmental Entomology*, 29, 35–42.

Snyder, W.E., Snyder, G.B., Finke, D.L. & Straub, C.S. (2006). Predator biodiversity strengthens herbivore suppression. *Ecology Letters*, 9, 789–796.

Sokol-Hessner, L. & Schmitz, O.J. (2002). Aggregate Effects of Multiple Predator Species on a Shared Prey. *Ecology*, 83, 2367-2372

Werling, B.P., Lowenstein, D.M., Straub, C.S. & Gratton, C. (2012). Multi-predator effects produced by functionally distinct species vary with prey density. *Journal of Insect Science*, 12, 1-7

Wilby, A., Anglin, L.A. & Nesbit, C.M. (2013). Plant species composition alters the sign and strength of an emergent multi-predator effect by modifying predator foraging behaviour. *Plos One*, 8, e70258.

Wilby, A. & Orwin, K.H. (2013). Herbivore species richness, composition and community structure mediate predator richness effects and top-down control of herbivore biomass. *Oecologia*, 172, 1167–1177.

Wilby, A., Villareal, S.C., Lan, L.P., Heong, K.L. & Thomas, M.B. (2005). Functional benefits of predator species diversity depend on prey identity. *Ecological Entomology*, 30, 497–501.

Wiethoff, J., Poehling, H.M. & Meyhofer, R. (2004). Combining plant- and soil-dwelling predatory mites to optimise biological control of thrips. *Experimental and Applied Acarology*, 34, 239–261.

Yasuda, H. & Kimura, T. (2001). Interspecific interactions in a tri-trophic arthropod system: effects of a spider on the survival of larvae of three predatory ladybirds in relation to aphids. *Experimental and Applied Acarology.*, 98, 17–25.
