# Supplementary material for: Functional diversity positively affects prey suppression by invertebrate predators: a meta‐analysis
Source: Ecology. 2018 Jul 5;99(8):1771–82. doi: 10.1002/ecy.2378 (PMC6099248; doi:10.1002/ecy.2378)
Supplement: Supplementary file 3 [file ECY-99-1771-s003.docx]

| **Trait** | **Categories** | **Definition** |
| --- | --- | --- |
| Habitat domain | Foliar | Predator species that predominantly hunt on plant foliage. Example Coccinellidae and Miridae. |
|  | Ground or base of plant (BPG) | Predators that predominantly hunt on the ground or around the base of plant. Example Carabidae. |
|  | Broad | Predators that are likely to hunt in both foliar and ground domains. Examples Lycosidae and Phalangiidae. |
| Hunting strategy | Sit and wait (SW) | Predator species waits for prey as opposed to actively pursuing prey. Examples Nabis species. |
|  | Ambush and pursue (AP) | Predator species waits for prey and then actively pursues once a prey item has been identified. Example Misumenops species. |
|  | Active | Predator actively searches and pursues prey. Example Cocinnellidae. |
| Diet breadth | Generalist | Broad arthropod diet with little or no feeding specialisation documented for a particular herbivore species. Example Lycosidae. |
|  | Specialist | Specialisation documented for particular herbivore species, however this categorisation does not preclude intraguild predation or alternate prey species. This category also includes parasitoid species. Example Phytoseiulus. |
|  |  |  |
| Body size (mm) |  | Mean body length across the life stage of the predator species in mm. |

**Appendix S3**

Table of species functional traits and their definitions. Also shown are the trait categorisations for each of the species included in the meta-analysis.

**Table S1.** Species functional trait categories and their definitions.

**Table S2.** All the species included in the studies used in the meta-analysis; their code used in analysis; trait categorisations for diet breadth, hunting strategy and habitat domain; mean body size (mm); and sources used for trait information.

| **Predator** | **Code** | **Diet breadth** | **Habitat domain** | **Hunting strategy** | **Size (mm)** | **Ref** |
| --- | --- | --- | --- | --- | --- | --- |
| *Adalia bipunctata (adult)* | Ab_a | Specialist | Foliar | Active | 4.5 | Agarwala, B.K. and Dixon, A.F. (1993). Kin recognition: egg and larval cannibalism in Adalia bipunctata (Coleoptera: Coccinellidae). *Eur. J. Entomol.*, **90,**.45-50.  Pervez, A. (2005). Ecology of two‐spotted ladybird, Adalia bipunctata: a review. *J. Appl. Entomol.*, **129***,*  465-474. |
| *Adalia bipunctata (larvae)* | Ab_l | Specialist | Foliar | Active | 3.25 | Agarwala, B.K. and Dixon, A.F. (1993). Kin recognition: egg and larval cannibalism in Adalia bipunctata (Coleoptera: Coccinellidae). *Eur. J. Entomol.*, **90,**.45-50.  Pervez, A. (2005). Ecology of two‐spotted ladybird, Adalia bipunctata: a review. *J. Appl. Entomol*, **129***,*  465-474. |
| *Amblyseius fallacis* | Af | Specialist | Foliar | Active | 0.5 | Appliedbio-nomics. (2017). *Amblyseius* (Neoseiulus) *fallacis*. [online] Available at: <https://www.appliedbio-nomics.com/wp-content/uploads/201-fallacis.pdf>. [Accessed 4 Jul. 2017].  Hogmire, H. (1995). *Mid-Atlantic orchard monitoring guide*. Ithaca, N.Y. Northeast Regional Agricultural Engineering Service, Cooperative Extension. |
| *Amblyseius cucumeris* | Ac | Specialist | Foliar | Active | 0.4 | Evergreen Growers Supply. (2017). Amblyseius cucumeris. [online] Available at: https://www.evergreengrowers.com/thrips-control/amblyseius-cucumeris-thrips-control/amblyseius-cucumeris.html [Accessed 4 Jul. 2017].  Wiethoff, J., Poehling, H.M. & Meyhofer, R. (2004). Combining plant- and soil-dwelling predatory mites to optimise biological control of thrips. *Experimental and Applied Acarology*, 34, 239–261. |
| *Anthocoris nemorum (adult)* | An_a | Generalist | Foliar | Active | 3.5 | Meyling, N.V., Enkegaard, A. and Brødsgaard, H. (2004). Intraguild predation by Anthocoris nemorum (Heteroptera: Anthocoridae) on the aphid parasitoid Aphidius colemani (Hhymenoptera: Braconidae). *Biocontrol Sci.Techn*, **14**, 627-630.  Sigsgaard, L. (2010). Habitat and prey preferences of the two predatory bugs Anthocoris nemorum (L.) and A. nemoralis (Fabricius) (Anthocoridae: Hemiptera-Heteroptera). *Biol.l Control.*, **53**, 46-54. |
| *Anyphaena pacifica (juvenile)* | Ap | Generalist | Broad | Active | 4.2 | [Hogg, B.N. and Daane, K.M. (2014). The roles of top and intermediate predators in herbivore suppression: contrasting results from the field and laboratory. *Ecol. Entomol.*, **39**, 49-158.](http://onlinelibrary.wiley.com/doi/10.1111/een.12079/full;) |
| *Aphidius ervi* | Ae | Specialist | Foliar | Active | 2.5 | Applied Bio-nomics. (2017). Aphidius (Aphidius matricariae, A. colemani, A. ervi) Aphid Parasites. [online] Available at: http://www.appliedbio-nomics.com/wp-content/uploads/242-aphidius.pdf [Accessed 4 Jul. 2017]. |
| *Aphidius floridaensis (adult)* | Aflor | Specialist | Foliar | Active | 2.5 | Ferguson, K.I. and Stiling, P. (1996). Non-additive effects of multiple natural enemies on aphid populations. *Oecologia*, **108**, 375-379. |
| *Aphidius matricariae* | Amat | Specialist | Foliar | Active | 2.5 | Applied Bio-nomics. (2017). Aphidius (Aphidius matricariae, A. colemani, A. ervi) Aphid Parasites. [online] Available at: http://www.appliedbio-nomics.com/wp-content/uploads/242-aphidius.pdf [Accessed 4 Jul. 2017]. |
| *Aphidoletes aphidimyza (larvae)* | Aaphi | Specialist | Foliar | Active | 2.5 | Cornell University College of Agriculture and Life Science. (2017). Aphidoletes aphidimyza. [online] Available at: https://biocontrol.entomology.cornell.edu/predators/Aphidoletes.php [Accessed 4 Jul. 2017]. |
| *Atypena formosana (juvenile)* | Afor | Generalist | Broad | SW | 3 | Sigsgaard, L. (2007). Early season natural control of the brown planthopper, Nilaparvata lugens: the contribution and interaction of two spider species and a predatory bug. *B. Entomol. Res.*, **97**, 533-544.  Sigsgaard, L., Toft, S. and Villareal, S. (2001). Diet‐dependent fecundity of the spiders Atypena formosana and Pardosa pseudoannulata, predators in irrigated rice. *Agr. Forest Entomol.*, **3**, 285-295. |
| *Calathus fuscipes* | Cf | Generalist | BPG | Active | 12 | Expert opinion. |
| *Cheiracanthium mildei (juvenile)* | Cm | Generalist | Broad | Active | 5.17 | Hogg, B.N. and Daane, K.M. (2011). Diversity and invasion within a predator community: impacts on herbivore suppression. *Journal of Applied Ecology*, **48**, 453-461.  Spiders.us. (2017). Cheiracanthium mildei (Longlegged Sac Spider) Pictures and Spider Identification. [online] Available at: http://www.spiders.us/species/cheiracanthium-mildei/ [Accessed 4 Jul. 2017]. |
| *Chrysoperla carnea (larvae)* | Cc_l | Specialist | Foliar | Active | 4.85 | Hanskumar, S.V. (2012). *Feeding potential and insecticidal safety evaluation of Chrysoperla sp.(carnea-group)* (Doctoral dissertation, Iari, Division Of Entomology).  Mochizuki, A., Naka, H., Hamasaki, K. and Mitsunaga, T. (2006). Larval cannibalism and intraguild predation between the introduced green lacewing, Chrysoperla carnea, and the indigenous trash-carrying green lacewing, Mallada desjardinsi (Neuroptera: Chrysopidae), as a case study of potential nontarget effect assessment. *Environ. Entomol.*, **35**, 1298-1303.  Ulhaq, M.M., Sattar, A., Salihah, Z., Farid, A., Usman, A. and Khattak, S.U.K. (2006). Effect of different artificial diets on the biology of adult green lacewing (Chrysoperla carnea Stephens). *Songklanakarin J Sci Technol*, **28**, 1-8. |
| *Chrysoperla plorabunda (larvae)* | Cp_l | Specialist | Foliar | Active | 4.85 | Hanskumar, S.V. (2012). *Feeding potential and insecticidal safety evaluation of Chrysoperla sp.(carnea-group)* (Doctoral dissertation, Iari, Division Of Entomology).  Mochizuki, A., Naka, H., Hamasaki, K. and Mitsunaga, T. (2006). Larval cannibalism and intraguild predation between the introduced green lacewing, Chrysoperla carnea, and the indigenous trash-carrying green lacewing, Mallada desjardinsi (Neuroptera: Chrysopidae), as a case study of potential nontarget effect assessment. *Environ. Entomol.*, **35**, 1298-1303.  Ulhaq, M.M., Sattar, A., Salihah, Z., Farid, A., Usman, A. and Khattak, S.U.K. (2006). Effect of different artificial diets on the biology of adult green lacewing (Chrysoperla carnea Stephens). *Songklanakarin J Sci Technol*, **28**, 1-8. |
| *Clubiona saltitans* | Csal | Generalist | Broad | Active | 7.55 | Finke, D.L. and Denno, R.F. (2005). Predator diversity and the functioning of ecosystems: the role of intraguild predation in dampening trophic cascades. *Ecol. Lett.*, **8**, 1299-1306. |
| *Coccinella septempunctata (adult)* | Csem_a | Specialist | Foliar | Active | 7.6 | Cornell University College of Agriculture and Life Science. (2017). Coccinella septempunctata. [Online]. [4 July 2017]. Available from: <https://biocontrol.entomology.cornell.edu/predators/Coccinella.php> Accessed 4 Jul. 2017]. |
| *Coccinella septempunctata (larvae)* | Csem_l | Specialist | Foliar | Active | 5.5 | Cornell University College of Agriculture and Life Science. (2017). Coccinella septempunctata. [Online]. [4 July 2017]. Available from: <https://biocontrol.entomology.cornell.edu/predators/Coccinella.php> Accessed 4 Jul. 2017]. |
| *Coleomagilla maculata (adult)* | Cmac_a | Specialist | Foliar | Active | 5.5 | Cornell University College of Agriculture and Life Science. (2017). Coleomegilla maculata. [Online]. [4 July 2017]. Available from: <https://biocontrol.entomology.cornell.edu/predators/Coleomegilla.php>. [Accessed 4 Jul. 2017]. |
| *Cycloneda sanguinea (adult)* | Csang | Specialist | Foliar | Active | 4.75 | Gordon, R. D. (1985). The Coccinellidae (Coleoptera) of America North of Mexico Journal of the New York Entomological Society, Vol. **93**  Işıkber, A.A. and Copland, M.J.W., 2002. Effects of various aphid foods on Cycloneda sanguinea. *Entomol. Exp. Appl.*, **102**, 93-97. |
| *Cyclotrachelus sodalis* | Csod | Generalist | BPG | Active | 15 | Snyder, W.E. and Wise, D.H. (2000). Antipredator behavior of spotted cucumber beetles (Coleoptera: Chrysomelidae) in response to predators that pose varying risks. *Environ. Entomol.*, **29**, 35-42. |
| *Cyrtorhinus lividipennis (adult)* | Cl_a | Specialist | Foliar | Active | 2.85 | Wilby, A., Villareal, S.C., Lan, L.P., Heong, K.L. & Thomas, M.B. (2005). Functional benefits of predator species diversity depend on prey identity. Ecological Entomology, 30, 497–501. |
| *Diaeretiella rapae* | Dr | Specialist | Foliar | Active | 2.15 | Kant, R., Minor, M.A. and Trewick, S.A. (2012). Fitness gain in a koinobiont parasitoid Diaeretiella rapae (Hymenoptera: Aphidiidae) by parasitising hosts of different ages. *J. Asia-Pacific Entomol.*, **15**, 83-87.  Karad, N.K., Korat, D.M. (2014). Biology and morphometry of Diaeretiella rapae (Mclntosh) - a parasitoid of aphids*. Karnataka J. Agric. Sci., **27,** 531-533 |
| *Dicyphus tamaninii (nymph)* | Dt | Generalist | Foliar | Active | 4.5 | Agustí, N., Gabarra, R. (2009). Effect of adult age and insect density of Dicyphus tamaninii Wagner (Heteroptera: Miridae) on progeny. *J. Pest Sci.,* **82**, 241–246.  Wheeler, A. G. (2000). Predacious plant bugs (Miridae),. In C. W. Scaefer and A. R. Panizzi (eds.), Heteroptera of economic importance. CRC press, Boca Raton, FL. p 657–693 |
| *Episyrphus balteatus (larvae)* | Eb | Specialist | Foliar | Active | 15 | Biopol. (2017). Episyrphus balteatus. [online] Available at: http://www.biopol.nl/en/solutions/biological-pest-control/aphids/hoverfly/episyrphus-balteatus/ [Accessed 4 Jul. 2017]. |
| *Erigone atra* | Ea | Generalist | BPG | SW | 2.25 | Dinter, A. (2002). Microcosm studies on intraguild predation between female erigonid spiders and lacewing larvae and influence of single versus multiple predators on cereal aphids. *Journal of Applied Entomology*, **126**, 249-257.  Expert opinion.  Harvey, P.R., Nellist, D.R. & Telfer, M.G. (eds) 2002. Provisional atlas of British spiders (Arachnida, Araneae), Volumes 1 & 2. Huntingdon: Biological Records Centre. |
| *Forficula auricularia* | Fa | Generalist | Broad | Active | 13.5 | Department of Entomology (Penn State University). (2017). European Earwigs (Department of Entomology). [online] Available at: http://ento.psu.edu/extension/factsheets/earwigs [Accessed 4 Jul. 2017]. |
| *Geocoris pallens and Geocoris punctipes* (adult)* | Geo | Generalist | Foliar | Active | 4 | Bao‐Fundora, L., Ramirez‐Romero, R., Sánchez‐Hernández, C.V., Sánchez‐Martínez, J. and Desneux, N. (2016). Intraguild predation of Geocoris punctipes on Eretmocerus eremicus and its influence on the control of the whitefly Trialeurodes vaporariorum. *Pest Manag. Sci.*, **72**, 1110-1116.  Utah Pests Fact Sheet. (2011). Beneficial True Bugs: Big-Eyed Bugs. [online] Available at: http://extension.usu.edu/files/publications/factsheet/big-eyed-bugs.pdf [Accessed 4 Jul. 2017]. |
| *Grammonota trivitatta* | Gt | Generalist | BPG | SW | 3 | Denno, R.F., Mitter, M.S., Langellotto, G.A., Gratton, C. and Finke, D.L. (2004). Interactions between a hunting spider and a web‐builder: consequences of intraguild predation and cannibalism for prey suppression. *Ecol. Entomol.*, **29**, 566-577.  Wimp, G.M., Murphy, S.M., Lewis, D., Douglas, M.R., Ambikapathi, R., Van-Tull, L.A., Gratton, C. and Denno, R.F. (2013). Predator hunting mode influences patterns of prey use from grazing and epigeic food webs. *Oecologia*, **171,**1-11 |
| *Harmonia axyridis (adult)* | Haxy_a | Generalist | Foliar | Active | 6.75 | University of Michigan - Animal Diversity Web. (2017). Hippodamia convergens (convergent lady beetle). [online] Available at: http://animaldiversity.org/accounts/Hippodamia_convergens/ [Accessed 4 Jul. 2017]. |
| *Harmonia axyridis (larvae)* | Haxy_l | Generalist | Foliar | Active | 6.3 | University of Michigan - Animal Diversity Web. (2017). Hippodamia convergens (convergent lady beetle). [online] Available at: http://animaldiversity.org/accounts/Hippodamia_convergens/ [Accessed 4 Jul. 2017]. |
| *Harpalus pennsylvanicus (adult)* | Hpen | Generalist | BPG | Active | 14.5 | Department of Entomology (Penn State University). (2017). Ground and Tiger Beetles (Coleoptera: Carabidae) (Department of Entomology). [online] Available at: http://ento.psu.edu/extension/factsheets/ground-beetles [Accessed 4 Jul. 2017].  NC State University. (2017). The Ground Beetles of Eastern North Carolina Agriculture. [online] Available at: http://www4.ncsu.edu/~dorr/Insects/Predators/Ground_Beetle/Ground_Beetles1_final.pdf [Accessed 4 Jul. 2017]. |
| *Hippodamia convergens (adult)* | Hc_a | Specialist | Foliar | Active | 6 | University of Florida Entomolgy and Nematology. (2017). convergent ladybug - Hippodamia convergens. [online] Available at: http://entnemdept.ufl.edu/creatures/BENEFICIAL/convergent_lady_beetle.html [Accessed 4 Jul. 2017].  University of Michigan - Animal Diversity Web. (2017). Hippodamia convergens (convergent lady beetle). [online] Available at: http://animaldiversity.org/accounts/Hippodamia_convergens/ [Accessed 4 Jul. 2017]. |
| *Hippodamia convergens (larvae)* | Hc_l | Specialist | Foliar | Active | 5.5 | University of Florida Entomolgy and Nematology. (2017). convergent ladybug - Hippodamia convergens. [online] Available at: http://entnemdept.ufl.edu/creatures/BENEFICIAL/convergent_lady_beetle.html [Accessed 4 Jul. 2017].  University of Michigan - Animal Diversity Web. (2017). Hippodamia convergens (convergent lady beetle). [online] Available at: http://animaldiversity.org/accounts/Hippodamia_convergens/ [Accessed 4 Jul. 2017]. |
| *Hippodamia sinuata (larvae)* | Hs_l | Specialist | Foliar | Active | 5.5 | PDF at <http://mint.ippc.orst.edu/ladybeetfact.pdf> modified from: Berry, R., Hall, B., Mooney, P. and Delaney, D. (1998). Insects and Mites of Economic Importance in the Northwest. 2^nd^ ed. Corvallis, Or. Dept. of Entomology, Oregon State University |
| *Hippodamia tredecimpunctata (larvae)* | Ht_l | Specialist | Foliar | Active | 5.45 | Chinery, M., 1986. Collins guide to the insects of Britain and western Europe. London: Collins. p 258 |
| *Hippodamia variegata (larvae)* | Hv_l | Specialist | Foliar | Active | 4 | Farhadi, R., Allahyari, H. and Juliano, S.A. (2010). Functional response of larval and adult stages of Hippodamia variegata (Coleoptera: Coccinellidae) to different densities of Aphis fabae (Hemiptera: Aphididae). *Environ. Entomol.*, **39**, 1586-1592.  Rebolledo, R., Sheriff, J., Parra, L. and Aguilera, A., 2009. Life, seasonal cycles, and population fluctuation of Hippodamia variegata (Goeze)(coleoptera: coccinellidae), in the Central plain of La Araucanía region, Chile. *Chilean J. Agr. Res.*, **69**, 292-298. |
| *Hogna helluo* | Hh | Generalist | BPG | Active | 19.5 | Expert opinion.  Snyder, W.E. & Wise, D.H. (2001). Antipredator behavior of spotted cucumber beetles (Coleoptera : Chrysomelidae) in response to predators that pose varying risks. *Environmental Entomology*, **29**, 35–42. |
| *Hypoaspis aculeifer* | Hacul | Specialist | BPG | Active | 0.6 | Biological Services. (2017). Killer mites (Hypoaspis aculeifer) – Biological Services, Australia. [online] Available at: http://www.biologicalservices.com.au/products/killer-mites-23.html [Accessed 4 Jul. 2017].  Wiethoff, J., Poehling, H.M. & Meyhofer, R. (2004). Combining plant- and soil-dwelling predatory mites to optimise biological control of thrips. *Experimental and Applied Acarology*, 34, 239–261. |
| *Laricobius nigrinus* | Lnig | Specialist | Foliar | Active | 3 | Cornell Chronicle. (2017). *Cornell releases predator beetle to battle hemlock pest \| Cornell Chronicle*. [online] Available at: http://news.cornell.edu/stories/2009/11/cornell-releases-predator-beetle-battle-hemlock-pest [Accessed 4 Jul. 2017].  Zilahi-Balogh, G.M.G., Humble, L.M., Kok, L.T. and Salom, S.M. (2006). Morphology of Laricobius nigrinus (Coleoptera: Derodontidae), a predator of the hemlock woolly adelgid. *Canadian Entomol.*, **138**, 595-601. |
| *Laricobius nigrinus (larvae)* | Lnig_l | Specialist | Foliar | Active | 2.69 | Cornell Chronicle. (2017). *Cornell releases predator beetle to battle hemlock pest \| Cornell Chronicle*. [online] Available at: http://news.cornell.edu/stories/2009/11/cornell-releases-predator-beetle-battle-hemlock-pest [Accessed 4 Jul. 2017].  Zilahi-Balogh, G.M.G., Humble, L.M., Kok, L.T. and Salom, S.M. (2006). Morphology of Laricobius nigrinus (Coleoptera: Derodontidae), a predator of the hemlock woolly adelgid. *Canadian Entomol.*, **138**, 595-601. |
| *Macrolophus caliginosus* | Mc | Generalist | Foliar | Active | 3.25 | Bonato, O., Couton, L. and Fargues, J. (2006). Feeding preference of Macrolophus caliginosus (Heteroptera: Miridae) on Bemisia tabaci and Trialeurodes vaporariorum (Homoptera: Aleyrodidae). *J. Econ. Entomol.*, **99**, 1143-1151.  Lucas, E. and Alomar, O. (2001). Macrolophus caliginosus (Wagner) as an intraguild prey for the zoophytophagous Dicyphus tamaninii Wagner (Heteroptera: Miridae). *Biol. Control*, **20**, 147-152. |
| *Marpissa pikei* | Mpik | Generalist | Foliar | Active | 8 | Expert opinion.  Brodeur, J. and Boivin, G. eds., 2006. *Trophic and guild interactions in biological control.* New York: Springer. p 249 |
| *Meteorus ictericus* | Mict | Specialist | Foliar | Active | 5.15 | Bürgi, L.P. and Mills, N.J. (2013). Developmental strategy and life history traits of Meteorus ictericus, a successful resident parasitoid of the exotic light brown apple moth in California. *Biol. Control*, **66**, 173-182. |
| *Metioche vittaticollis* | Mvit | Specialist | Foliar | Active | 10 | Expert opinion.  Wilby, A., Villareal, S.C., Lan, L.P., Heong, K.L. and Thomas, M.B., 2005. Functional benefits of predator species diversity depend on prey identity. *Ecol. Entomol.*, **30**, 497-501. |
| *Micraspis crocea (adult)* | Mcroc | Specialist | Foliar | Active | 4.5 | Shanker, C., Mohan, M., Sampathkumar, M., Lydia, C. and Katti, G., 2013. Functional significance of Micraspis discolor (F.)(Coccinellidae: Coleoptera) in rice ecosystem. *J. Appl. Entomol.*, **137**, 601-609.  Shepard, B.M. and Rapusas, H.R. (1989). Life cycle of Micraspis sp. on brown planthopper (BPH) and rice pollen. *International Rice Research Newsletter*  *(Philippines)*. |
| *Misumenops (two mid instar)* | Mis | Generalist | Foliar | AP | 6 | Expert opinion.  Yasuda, H. & Kimura, T. (2001). Interspecific interactions in a tri-trophic arthropod system: effects of a spider on the survival of larvae of three predatory ladybirds in relation to aphids. Experimental and Applied Acarology., 98, 17–25 |
| *Misumenops tricuspidatus* | Mtric | Generalist | Foliar | AP | 6 | Expert opinion.  Yasuda, H. & Kimura, T. (2001). Interspecific interactions in a tri-trophic arthropod system: effects of a spider on the survival of larvae of three predatory ladybirds in relation to aphids. Experimental and Applied Acarology., 98, 17–25 |
| *Nabis (sp)* | Nabis | Generalist | Foliar | SW | 7.5 | Aquilino, K.M., Cardinale, B.J. & Ives, A.R. (2005). Reciprocal effects of host plant and natural enemy diversity on herbivore suppression: an empirical study of a model tritrophic system. *Oikos*, **108**, 275–282.  Berry, R., Hall, B., Mooney, P. and Delaney, D. (1998). Insects and Mites of Economic Importance in the Northwest. 2^nd^ ed. Corvallis, Or.: Dept. of Entomology, Oregon State University |
| *Nabis alternatus* | Nalt | Generalist | Foliar | SW | 8 | Northfield, T.D., Snyder, G.B., Ives, A.R. & Snyder, W.E. (2010). Niche saturation reveals resource partitioning among consumers. *Ecology Letters*, **13**, 338–348  Berry, R., Hall, B., Mooney, P. and Delaney, D. (1998). Insects and Mites of Economic Importance in the Northwest. 2^nd^ ed. Corvallis, Or.: Dept. of Entomology, Oregon State University |
| *Naemia seriata* | Nser | Generalist | Foliar | Active | 5.35 | Matsumura, M., Trafelet-Smith, G.M., Gratton, C., Finke, D.L., Fagan, W.F. and Denno, R.F. (2004). Does intraguild predation enhance predator performance? A stoichiometric perspective. *Ecology*, **85**, 2601-2615.  Marriott, S.M., Giberson, D.J. and McCorquodale, D.B., (2009). Changes in the status and geographic ranges of Canadian Lady Beetles (Coccinellinae) and the selection of candidates for risk assessment. Part 1. Foundation Report. |
| *Nesidiocoris tenuis (nymph)* | Nten | Generalist | Foliar | Active | 2.5 | Biological Services. (2017). Nesidiocoris (Nesidiocoris tenuis) – Biological Services, Australia. [online] Available at: http://www.biologicalservices.com.au/products/nesidiocoris-28.html [Accessed 4 Jul. 2017].  Gervassio, S., Nadia, G., Pérez‐Hedo, M., Luna, M.G. and Urbaneja, A. (2016). Intraguild predation and competitive displacement between Nesidiocoris tenuis and Dicyphus maroccanus, 2 biological control agents in tomato pests. *Insect Sci.*, doi:10.1111/1744-7917.12361 |
| *Nesticodes rufipes* | Nest | Generalist | Foliar | SW | 2 | Rosenheim, J.A., Limburg, D.D., Colfer, R.G., Fournier, V., Hsu, C.L., Leonardo, T.E. & Nelson, E.H. (2004a) Herbivore population suppression by an intermediate predator, Phytoseiulus macropilis, is insensitive to the presence of an intraguild predator: an advantage of small body size? *Oecologia*, 140, 577–585. 2)  Rosenheim, J.A., Glik, T.E., Goeriz, R.E. & Rämert, B. (2004b) Linking a predator’s foraging behavior with its effects on herbivore population suppression. *Ecology*, 85, 3362–3372. |
| *Oedothorax apicatus* | Oapi | Generalist | BPG | SW | 2.75 | Dinter, A. (2002). Microcosm studies on intraguild predation between female erigonid spiders and lacewing larvae and influence of single versus multiple predators on cereal aphids. *Journal of Applied Entomology*, **126**, 249-257.  Spider and Harvestman Recording Scheme website. (2017). Summary for Oedothorax apicatus (Araneae). [online] Available at: http://srs.britishspiders.org.uk/portal/p/Summary/s/Oedothorax+apicatus [Accessed 4 Jul. 2017]. |
| *Oligota sp.* | Oli | Specialist | Foliar | Active | 0.5 | Rosenheim, J.A., Limburg, D.D., Colfer, R.G., Fournier, V., Hsu, C.L., Leonardo, T.E. & Nelson, E.H. (2004a) Herbivore population suppression by an intermediate predator, Phytoseiulus macropilis, is insensitive to the presence of an intraguild predator: an advantage of small body size? *Oecologia*, **140**, 577–585.  Rosenheim, J.A., Glik, T.E., Goeriz, R.E. & Rämert, B. (2004b) Linking a predator’s foraging behavior with its effects on herbivore population suppression. *Ecology*, **85**, 3362–3372.  Williams, S.A. (1976). The genus oligota (Coleoptera: Staphylinidae) in New Zealand. *New Zealand Journal of Zoology*, **3**, 247-255 |
| *Orius tristicolor (adult)* | Otri | Generalist | Foliar | Active | 3 | Cornell University College of Agriculture and Life Science. (2017). *Orius tristicolor and O. insidiosus*. [online] Available at: https://biocontrol.entomology.cornell.edu/predators/Orius.php [Accessed 4 Jul. 2017]. |
| *Orthotylus marginali* | Omarg | Generalist | Foliar | SW | 6.4 | Bantock, T. (2017). (Miridae) Orthotylus marginalis. [online] Britishbugs.org.uk. Available at: https://www.britishbugs.org.uk/heteroptera/Miridae/orthotylus_marginalis.html [Accessed 4 Jul. 2017].  Björkman, C. and Liman, A.S. (2005). Foraging behaviour influences the outcome of predator–predator interactions. *Ecol. Entomol.*, **30**, 164-169. |
| *Pardosa littoralis (adult)* | Pl_a | Generalist | Broad | Active | 4 | Finke, D.L. and Denno, R.F. (2005). Predator diversity and the functioning of ecosystems: the role of intraguild predation in dampening trophic cascades. *Ecol. Letters*, **8**, 1299-1306.  Lewis, D. and Denno, R.F. (2009). A seasonal shift in habitat suitability enhances an annual predator subsidy. *J. Anim. Ecol.*, 78, 752-760. |
| *Pardosa pseudoannulata* | Pp | Generalist | BPG | Active | 8.5 | A Guide to Common Singapore Spiders. (2017). pond wolf spider (pardosa pseudoannulata). [online] Available at: http://habitatnews.nus.edu.sg/guidebooks/spiders/text/Pardosa_pseudoannulata.htm [Accessed 4 Jul. 2017].  Heong, K.L., Bleih, S. and Rubia, E.G. (1991). Prey preference of the wolf spider, Pardosa pseudoannulata (Boesenberg et Strand). *Res. Popul. Ecol*., **33**, 179-186. |
| *Phidippus rimator* | Prim | Generalist | Broad | Active | 8.7 | Sokol-Hessner, L. and Schmitz, O.J. (2002). Aggregate effects of multiple predator species on a shared prey. *Ecology*, **83**, 2367-2372.  Horton, C.C. (1983). Predators of two orb-web spiders (Araneae, Araneidae). *The Journal of Arachnology*, **11**, 447-449.  TGSpId: Table Grape Spider Identification. (2017). TGSpID-Factsheet Phidippus clarus. [online] Available at: http://itp.lucidcentral.org/id/table-grape/tgspid/html/fsheet_phidippus_clarus.htm [Accessed 4 Jul. 2017]. |
| *Philonthus sp (adult)* | Phil | Generalist | BPG | Active | 10.5 | Expert opinion. |
| *Phalangium opilio* | Popi | Generalist | Broad | AP | 3.75 | NatureSpot. (2017). Phalangium opilio - Phalangium opilio \| NatureSpot. [online] Available at: http://www.naturespot.org.uk/species/phalangium-opilio [Accessed 4 Jul. 2017].  Expert opinion. |
| *Phytoseiulus macropilis* | Pmacro | Specialist | Foliar | Active | 0.5 | Rosenheim, J.A., Limburg, D.D., Colfer, R.G., Fournier, V., Hsu, C.L., Leonardo, T.E. & Nelson, E.H. (2004). Herbivore population suppression by an intermediate predator, Phytoseiulus macropilis, is insensitive to the presence of an intraguild predator: an advantage of small body size? *Oecologia*, **140**, 577–585.  Okassa, M., Tixier, M.S. and Kreiter, S., 2010. Morphological and molecular diagnostics of Phytoseiulus persimilis and Phytoseiulus macropilis (Acari: Phytoseiidae). *Exp. Appl. Acarol.*, **52**, 291-303. |
| *Pisaurina mira* | Pmir | Generalist | Foliar | SW | 14 | University of Michigan - Animal Diversity Web. (2017). Pisaurina mira. [online] Available at: http://animaldiversity.org/accounts/Pisaurina_mira/ [Accessed 4 Jul. 2017].  Sokol-Hessner, L. and Schmitz, O.J. (2002). Aggregate effects of multiple predator species on a shared prey. *Ecology*, **83**, .2367-2372. |
| *Podisus maculiventris (adult)* | Pmac_a | Generalist | Foliar | Active | 10.75 | Cornell University College of Agriculture and Life Science. (2017). Podisus maculiventris. [online] Available at: https://biocontrol.entomology.cornell.edu/predators/Podisus.php [Accessed 4 Jul. 2017].  University of Florida Entomolgy and Nematology. (2017). Spined soldier bug - Podisus maculiventris Say. [online] Available at: http://entnemdept.ufl.edu/creatures/beneficial/podisus_maculiventris.htm [Accessed 4 Jul. 2017]. |
| *Podisus maculiventris (nymph)* | Pmac_n | Generalist | Foliar | Active | 5.65 | Cornell University College of Agriculture and Life Science. (2017). Podisus maculiventris. [online] Available at: https://biocontrol.entomology.cornell.edu/predators/Podisus.php [Accessed 4 Jul. 2017].  University of Florida Entomolgy and Nematology. (2017). Spined soldier bug - Podisus maculiventris Say. [online] Available at: http://entnemdept.ufl.edu/creatures/beneficial/podisus_maculiventris.htm [Accessed 4 Jul. 2017]. |
| *Propylea japonica (larvae)* | Pjap_l | Specialist | Foliar | Active | 4.5 | Ouyang, F., Men, X., Yang, B., Su, J., Zhang, Y., Zhao, Z. and Ge, F. (2012). Maize benefits the predatory beetle, Propylea japonica (Thunberg), to provide potential to enhance biological control for aphids in cotton. *PloS One*, **7**, p.e44379.  Zhang, S.Z., Zhang, F. and Hua, B.Z. (2007). Suitability of various prey types for the development of Propylea japonica (Coleoptera: Coccinellidae). *Eur. J. Entomol.*, **104**, 149. |
| *Psyllaephagus bliteus (adult)* | Pblit | Specialist | Foliar | Active | 1.54 | Daane, K.M., Sime, K.R., Dahlsten, D.L., Andrews, J.W. and Zuparko, R.L. (2005). The biology of Psyllaephagus bliteus Riek (Hymenoptera: Encyrtidae), a parasitoid of the red gum lerp psyllid (Hemiptera: Psylloidea). *Biol. Control*, **32**, 228-235. |
| *Pterostichus madidus* | Pmad | Generalist | BPG | Active | 17.5 | NatureSpot. (2017). Black Clock Beetle - Pterostichus madidus \| NatureSpot. [online] Available at: http://www.naturespot.org.uk/species/black-clock-beetle [Accessed 4 Jul. 2017].  Expert opinion. |
| *Pterostichus melanarius* | Pmel | Generalist | BPG | Active | 15 | NatureSpot. (2017). Pterostichus melanarius - Pterostichus melanarius \| NatureSpot. [online] Available at: http://www.naturespot.org.uk/species/pterostichus-melanarius [Accessed 4 Jul. 2017].  Expert opinion. |
| *Rabidosa rabida* | Rrab | Generalist | BPG | AP | 17 | University of Michigan - Animal Diversity Web. (2017). *Rabidosa rabida*. [online] Available at: http://animaldiversity.org/accounts/Rabidosa_rabida [Accessed 4 Jul. 2017].  Sokol-Hessner, L. and Schmitz, O.J. (2002). Aggregate effects of multiple predator species on a shared prey. *Ecology*, **83**, .2367-2372. |
| *Salticus scenicus* | Sscen | Generalist | Broad | Active | 7 | Drieu, R. and Rusch, A. (2016). Conserving species‐rich predator assemblages strengthens natural pest control in a climate warming context. *Agricultural and Forest Entomology*, 19, 52-59.  Arkive. (2017). Zebra spider - *Salticus scenicus* \| Arkive. [online] Available at: http://www.arkive.org/zebra-spider/salticus-scenicus/#text=All [Accessed 4 Jul. 2017]. |
| *Sasajiscymnus tsugae* | Stsu | Specialist | Foliar | Active | 0.48 | Cornell University College of Agriculture and Life Science. (2017). Sasajiscymnus tsugae. [online] Available at: https://biocontrol.entomology.cornell.edu/predators/sasajiscymnus.php [Accessed 4 Jul. 2017]. |
| *Sasajiscymnus tsugae (larvae)* | Stsu_l | Specialist | Foliar | Active | 1.9 | Cornell University College of Agriculture and Life Science. (2017). Sasajiscymnus tsugae. [online] Available at: https://biocontrol.entomology.cornell.edu/predators/sasajiscymnus.php [Accessed 4 Jul. 2017]. |
| *Stethorus siphonulus* | Ssiph | Specialist | Foliar | Active | 0.35 | Linking a predator’s foraging behavior with its effects on herbivore population suppression. Ecology, 85, 3362–3372.  Evergreen growers. (2017). Stethorus punctillum. [online] Available at: http://www.evergreengrowers.com/stethorus-punctillum-spider-mite-destroyer.html [Accessed 4 Jul. 2017].  Rosenheim, J.A., Limburg, D.D., Colfer, R.G., Fournier, V., Hsu, C.L., Leonardo, T.E. and Nelson, E.H. (2004). Herbivore population suppression by an intermediate predator, Phytoseiulus macropilis, is insensitive to the presence of an intraguild predator: an advantage of small body size? *Oecologia*, **140**, 577-585. |
| *Tachyporus hypnorum (adult)* | Thyp | Generalist | Broad | Active | 3.5 | NatureSpot. (2017). *Tachyporus hypnorum - Tachyporus hypnorum* \| *NatureSpot.* [online] Available at:  <http://www.naturespot.org.uk/species/tachyporus-hypnorum> [Accessed 4 Jul. 2017].  Petersen, M.K. (1997). Life histories of two predaceous beetles, Bembidion lampros and Tachyporus hypnorum, in the agroecosystem. Swedish University of Agricultural Sciences.  Vancouver. |
| *Theridion melanurum* | Tmel | Generalist | Broad | SW | 1.735 | Hogg, B.N. and Daane, K.M. (2011). Diversity and invasion within a predator community: impacts on herbivore suppression. *J. Appl. Ecol.*, **48**, 453-461.  NatureSpot. (2017). *Theridion melanurum - Theridion melanurum \| NatureSpot*. [online] Available at: http://www.naturespot.org.uk/species/theridion-melanurum [Accessed 4 Jul. 2017]. |
| *Typhlodromus pyri* | Tpy | Specialist | Foliar | Active | 0.6 | Washington State University - Orchard Pest Management Online. (2017). Typhlodromus occidentalis. [online] Available at: http://jenny.tfrec.wsu.edu/opm/displaySpecies.php?pn=830 [Accessed 4 Jul. 2017]. |
| *Tytthus vagus (adult)* | Tvag | Specialist | Foliar | Active | 2.34 | Finke, D.L. & Denno, R.F. (2002) Intraguild Predation Diminished in Complex-Structured Vegetation: Implications for Prey Suppression. *Ecology*, 83, 643.; 2  Henry, T.J. (2012). Revision of the Plant Bug Genus Tytthus (Hemiptera, Heteroptera, Miridae, Phylinae). *ZooKeys*, **220**, 1-114 |
| *Zelus renardii (adult)* | Zren_a | Generalist | Foliar | Active | 13.2 | Hart, E.R. (1986). Genus Zelus Fabricius in the United States, Canada, and Northern Mexico (Hemiptera: Reduviidae). Ann. Ent. Soc. Am. **79,** 535-548.  Thomas, H.J., Froeschner. R.C. (1988). Catalog of the Heteroptera, or True Bugs of Canada and the Continental United States. Brill Academic Publishers. Leiden, Netherlands.  Schaefer C.W., Panizzi A.R. (2000). Heteroptera of economic importance. CRC Press, Boca Raton, FL, 828. |
| *Zelus renardii (nymph)* | Zren_n | Generalist | Foliar | Active | 8.75 | Hart, E.R. (1986). Genus Zelus Fabricius in the United States, Canada, and Northern Mexico (Hemiptera: Reduviidae). *Ann. Ent. Soc. Am*. 79, 535-548.  Thomas, H.J., Froeschner. R.C. (1988). Catalog of the Heteroptera, or True Bugs of Canada and the Continental United States. Brill Academic Publishers. Leiden, Netherlands.  Schaefer C.W., Panizzi A.R. (2000). Heteroptera of economic importance. CRC Press, Boca Raton, FL, 828. |
