# Supplementary material for: Functional diversity positively affects prey suppression by invertebrate predators: a meta‐analysis
Source: Ecology. 2018 Jul 5;99(8):1771–82. doi: 10.1002/ecy.2378 (PMC6099248; doi:10.1002/ecy.2378)
Supplement: Supplementary file 4 [file ECY-99-1771-s004.docx]

**Appendix S4**

Table showing the collinearity between predator and prey body size variables (Table S1). Also shown is the model results where ratio_large_ (body size ratio between the largest predator and prey) was included instead of ratio_small_ (body size ratio between the smallest predator and prey).

**Table S1.** Pearson correlation coefficient between predator-prey size variables. Correlation where r >0.5 have been in highlighted in bold.

|  | Size of largest predator (mm)* | Size of smallest predator (mm)* | Size difference between predators | ratio_small_ | ratio_large_* | Prey size (mm) |
| --- | --- | --- | --- | --- | --- | --- |
| Size of largest predator (mm)* | 1.00 | **0.60** | **0.82** | -0.05 | 0.26 | 0.43 |
| Size of smallest predator (mm)* | **0.60** | 1.00 | 0.11 | -0.04 | -0.26 | **0.75** |
| Size difference between predators | **0.82** | 0.11 | 1.00 | 0.01 | 0.45 | 0.03 |
| ratio_small_ | -0.05 | -0.04 | 0.01 | 1.00 | **0.62** | -0.44 |
| ratio_large_* | 0.26 | -0.26 | 0.45 | **0.62** | 1.00 | -0.41 |
| Prey size (mm) | 0.43 | **0.75** | 0.03 | -0.44 | -0.41 | 1.00 |
| Parameters marked with * indicates variable was removed from analysis due to a high level of collinearity with other variables. | | | | | | |

ratio_small_ = body size ratio between the smallest predator and prey

ratio_large_ = body size ratio between the largest predator and prey

Size difference = mean pairwise distance in body size between the predator species

**Ratio_large_ models**

**Table S2**. 2AIC_c_ model subset for SMD_mean_ (predator polyculture compared to the mean of the component predator species in monocultures).

| **Rank** | **Model** | **AIC_c_** | **Weight** | **Relative weight** |
| --- | --- | --- | --- | --- |
| 1 | Functional diversity | 445.671 | 0.087 | 0.201 |
| 2 | Functional diversity + Size difference | 446.136 | 0.069 | 0.159 |
| 3 | Functional diversity + Phylogenetic diversity | 446.481 | 0.058 | 0.134 |
| 4 | Functional diversity + Phylogenetic diversity + Size difference | 447.097 | 0.043 | 0.099 |
| 5 | Functional diversity + Predator richness | 447.260 | 0.040 | 0.091 |
| 6 | Functional diversity + Prey richness | 447.378 | 0.037 | 0.086 |
| 7 | Functional diversity + ratio_large_ | 447.570 | 0.034 | 0.078 |
| 8 | Functional diversity + Predator richness + Size difference | 447.615 | 0.033 | 0.076 |
| 9 | Functional diversity + Prey size | 447.616 | 0.033 | 0.076 |

**Table S3**. Multimodel averaged parameter estimates for SMD_mean_ (predator polyculture compared to the mean of the component predator species in monocultures). Prey richness and predator richness estimate is the difference between the reference level (predator richness = 2 species and prey richness = 1 species). Parameter in bold indicate that the variable was included in the highest ranked model.

| **Parameter** | **Estimate** | **Importance** | **95% CI lower bound** | **95% CI upper bound** |
| --- | --- | --- | --- | --- |
| Prey size | -0.002 | 0.076 | -0.020 | 0.016 |
| ratio_large_ | -0.003 | 0.078 | -0.024 | 0.018 |
| Prey richness >1 | 0.010 | 0.086 | -0.045 | 0.065 |
| Predator richness >2 | 0.016 | 0.167 | -0.058 | 0.09 |
| Phylogenetic diversity | 0.102 | 0.233 | -0.287 | 0.491 |
| Size difference | -0.009 | 0.334 | -0.037 | 0.019 |
| **Functional diversity** | 0.452 | 1.000 | 0.070 | 0.834 |

**SMD_max_ ratio_large_ models**

**Table S4**. 2AIC_c_ model subset for SMD_max_ (predator polyculture compared to the most effective predator species in a monoculture).

| **Rank** | **Model** | **AIC_c_** | **Weight** | **Relative weight** |
| --- | --- | --- | --- | --- |
| 1 | Predator richness + Functional diversity | 543.920 | 0.086 | 0.242 |
| 2 | Predator richness + Functional diversity + Size difference | 544.817 | 0.055 | 0.154 |
| 3 | Predator richness + Functional diversity + ratio_large_ | 545.036 | 0.049 | 0.139 |
| 4 | Predator richness + Functional diversity + Phylogenetic diversity | 545.133 | 0.047 | 0.132 |
| 5 | Functional diversity | 545.170 | 0.046 | 0.130 |
| 6 | Functional diversity + ratio_large_ | 545.482 | 0.039 | 0.111 |
| 7 | Functional diversity + Size difference | 545.806 | 0.033 | 0.094 |

**Table S5**. Multimodel averaged parameter estimates for SMD_max_ (predator polyculture compared to the most effective predator species in a monoculture). Predator richness estimate is the difference between the reference level (predator richness = 2). Parameter in bold indicate that the variable was included in the highest ranked model.

| **Parameter** | **Estimate** | **Importance** | **95% CI lower bound** | **95% CI upper bound** |
| --- | --- | --- | --- | --- |
| Phylogenetic diversity | 0.052 | 0.132 | -0.180 | 0.282 |
| Size difference | -0.006 | 0.248 | -0.029 | 0.017 |
| ratio_large_ | -0.030 | 0.249 | -0.143 | 0.083 |
| **Predator richness >2** | -0.158 | 0.666 | -0.464 | 0.148 |
| **Functional diversity** | 0.471 | 1.000 | 0.057 | 0.885 |
