# Supplementary material for: Functional diversity positively affects prey suppression by invertebrate predators: a meta‐analysis
Source: Ecology. 2018 Jul 5;99(8):1771–82. doi: 10.1002/ecy.2378 (PMC6099248; doi:10.1002/ecy.2378)
Supplement: Supplementary file 5 [file ECY-99-1771-s005.docx]

**Appendix S5**

Funnel plots to assess publication bias.

Figure S1. Funnel plots for SMD_mean_ showing the effect size plotted against the a) standard error, b) sampling variance, c) inverse standard error and the d) inverse sampling variance for each point.

Figure S2. Funnel plots for SMD_max_ showing the effect size plotted against the a) standard error, b) sampling variance, c) inverse standard error and the d) inverse sampling variance for each point.


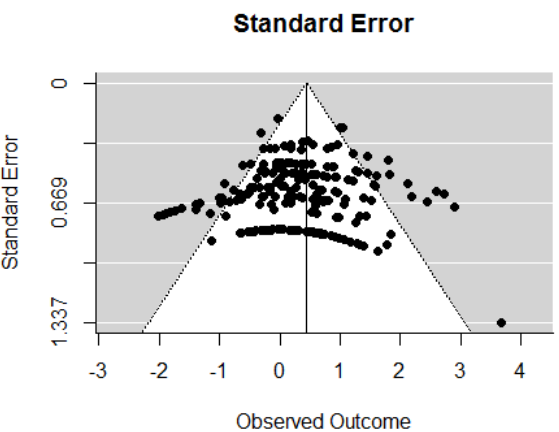


**S1a)**


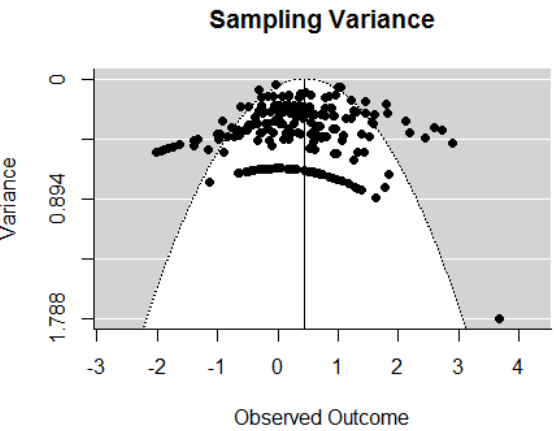


**S1b)**


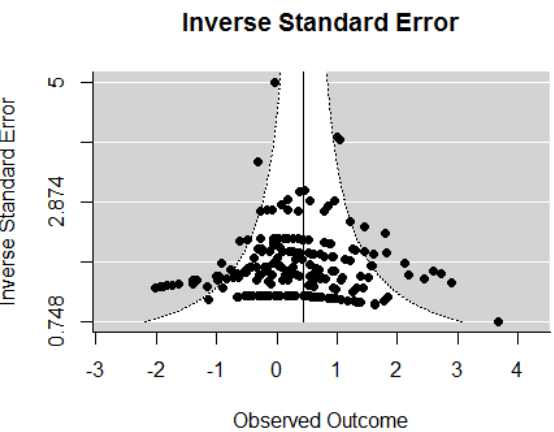


**S1c)**


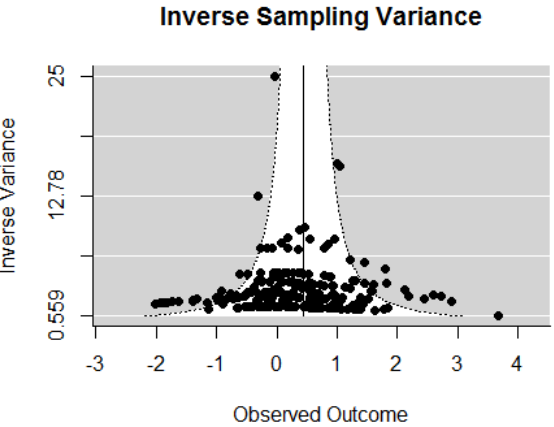


**S1d)**


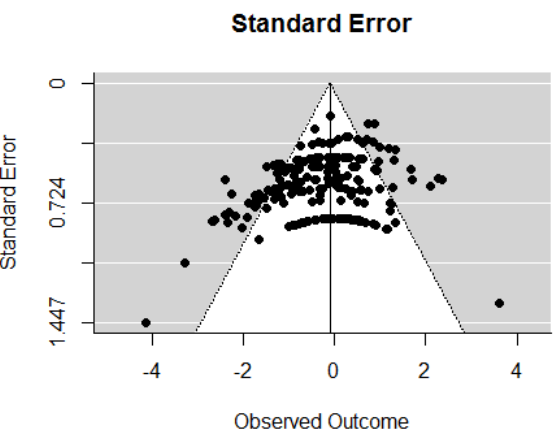


**S2a)**


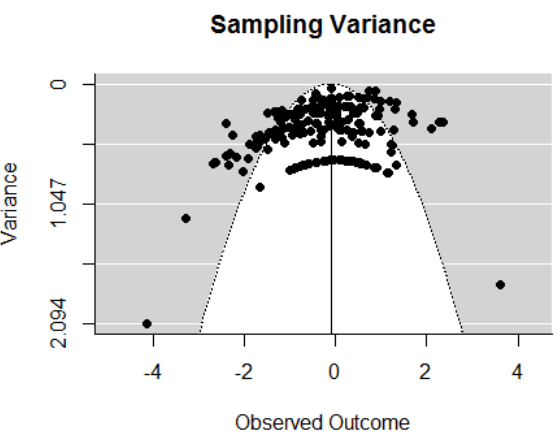


**S2b)**


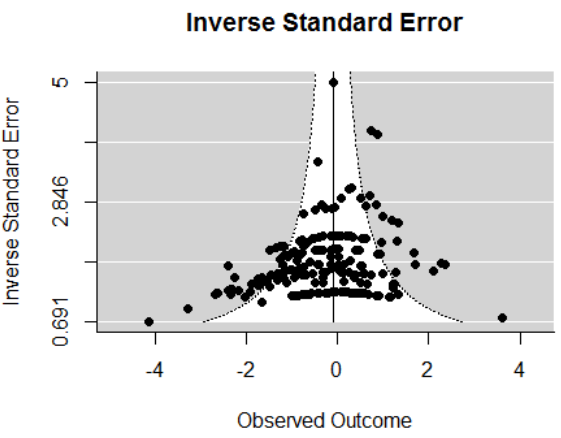


**S2c)**


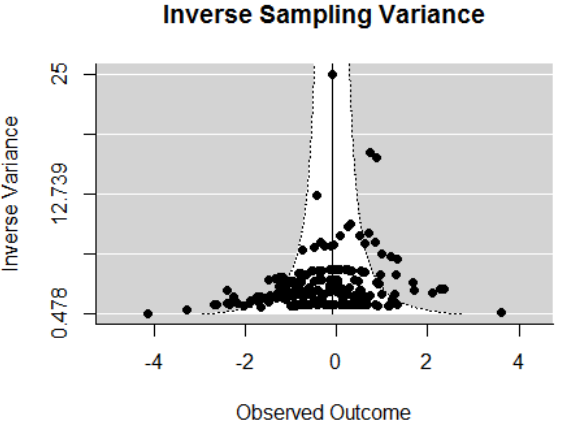


**S2d)**
