# Supplementary material for: Functional diversity positively affects prey suppression by invertebrate predators: a meta‐analysis
Source: Ecology. 2018 Jul 5;99(8):1771–82. doi: 10.1002/ecy.2378 (PMC6099248; doi:10.1002/ecy.2378)
Supplement: Supplementary file 6 [file ECY-99-1771-s006.docx]

**Appendix S6**

Model 2AIC_c_ subset for SMD_mean_ and SMD_max_ metrics.

**SMD_mean_**

**Table S1**. 2AIC_c_ model subset for SMD_mean_ (predator polyculture compared to the mean of the component predator species in monocultures)_._

| **Rank** | **Model** | **AIC_c_** | **Weight** | **Relative weight** |
| --- | --- | --- | --- | --- |
| 1 | Functional diversity | 445.671 | 0.070 | 0.145 |
| 2 | Functional diversity + Size difference | 446.136 | 0.055 | 0.115 |
| 3 | Functional diversity + ratio_small_ | 446.167 | 0.054 | 0.113 |
| 4 | Functional diversity + Phylogenetic diversity | 446.481 | 0.046 | 0.097 |
| 5 | Functional diversity + Size difference + ratio_small_ | 446.860 | 0.038 | 0.080 |
| 6 | Functional diversity + Prey size + ratio_small_ | 446.906 | 0.037 | 0.078 |
| 7 | Functional diversity + Phylogenetic diversity + Size difference | 447.097 | 0.034 | 0.071 |
| 8 | Functional diversity + Predator richness | 447.260 | 0.031 | 0.065 |
| 9 | Functional diversity + Phylogenetic diversity + ratio_small_ | 447.266 | 0.031 | 0.065 |
| 10 | Functional diversity + Prey richness | 447.378 | 0.030 | 0.062 |
| 11 | Functional diversity + Predator richness + Size difference | 447.615 | 0.026 | 0.055 |
| 12 | Functional diversity + Prey size | 447.616 | 0.026 | 0.055 |

**SMD_max_**

**Table S2**. 2AIC_c_ model subset for SMD_max_ (predator polyculture compared to the most effective predator species in a monoculture)_._

| **Rank** | **Model** | **AIC_c_** | **Weight** | | **Relative weight** | |
| --- | --- | --- | --- | --- | --- | --- |
| 1 | Predator richness + Functional diversity + ratio_small_ | 542.820 | | 0.090 | | 0.267 |
| 2 | Predator richness + Functional diversity + ratio_small_ + Prey size | 543.295 | | 0.071 | | 0.211 |
| 3 | Predator richness + Functional diversity | 543.920 | | 0.052 | | 0.154 |
| 4 | Predator richness + Functional diversity + Size difference + ratio_small_ | 544.029 | | 0.049 | | 0.146 |
| 5 | Predator richness + Functional diversity + Phylogenetic diversity + ratio_small_ | 544.398 | | 0.041 | | 0.121 |
| 6 | Predator richness + Functional diversity + Size difference | 544.817 | | 0.033 | | 0.099 |
