# Supplementary material for: Functional diversity positively affects prey suppression by invertebrate predators: a meta‐analysis
Source: Ecology. 2018 Jul 5;99(8):1771–82. doi: 10.1002/ecy.2378 (PMC6099248; doi:10.1002/ecy.2378)
Supplement: Supplementary file 7 [file ECY-99-1771-s007.docx]

**Appendix S7**

Analysis of the individual traits diet breadth, hunting strategy and habitat domain on SMD_mean_ (predator polyculture compared to the mean of the component predator species in monocultures) and SMD_max_ (predator polyculture compared to the most effective predator species in a monoculture). A functional diversity only model has also been included for comparison.

**SMD_mean_**

**Table S1**. 2AIC_c_ model subset for SMD_mean._

| **Rank** | **Model** | **AIC_c_** | **Weights** | **Relative weight** |
| --- | --- | --- | --- | --- |
| 1 | Diet breadth | 443.960 | 0.479 | 0.709 |
| 2 | Diet breadth + Hunting strategy | 445.743 | 0.197 | 0.291 |

**Table S2**. Multimodel averaged parameter estimates for SMD_mean._

| **Parameter** | **Estimate** | **Importance** | **95% CI lower bound** | **95% CI upper bound** |
| --- | --- | --- | --- | --- |
| Hunting strategy | 0.023 | 0.291 | -0.098 | 0.144 |
| Diet breadth | 0.371 | 1.000 | 0.096 | 0.646 |

**Table S3.** Functional diversity only model for SMD_mean._

AIC_c_ = 445.671

| **Parameter** | **Estimate** | **95% CI lower bound** | **95% CI upper bound** |
| --- | --- | --- | --- |
| Functional diversity | 0.453 | 0.072 | 0.831 |

**SMD_max_**

**Table S4**. 2AIC_c_ model subset for SMD_max._

| **Rank** | **Model** | **AIC_c_** | **Weights** | **Relative weight** |
| --- | --- | --- | --- | --- |
| 1 | Diet breadth | 547.266 | 0.220 | 0.278 |
| 2 | Hunting strategy | 547.864 | 0.163 | 0.206 |
| 3 | Diet breadth + Hunting strategy | 547.882 | 0.162 | 0.204 |
| 4 | Null model | 547.942 | 0.157 | 0.198 |
| 5 | Diet breadth + Habitat domain | 549.028 | 0.091 | 0.115 |

**Table S5**. Multimodel averaged parameter estimates for SMD_max._

| **Parameter** | **Estimate** | **Importance** | **95% CI lower bound** | **95% CI upper bound** |
| --- | --- | --- | --- | --- |
| Habitat domain | 0.012 | 0.115 | -0.058 | 0.082 |
| Hunting strategy | 0.084 | 0.410 | -0.17 | 0.338 |
| Diet breadth | 0.141 | 0.596 | -0.179 | 0.461 |

**Table S6.** Functional diversity only model for SMD_max._

AIC_c_ = 545.170

| **Parameter** | **Estimate** | **95% CI lower bound** | **95% CI upper bound** |
| --- | --- | --- | --- |
| Functional diversity | 0.458 | 0.051 | 0.865 |
