# Supplementary material for: Functional diversity positively affects prey suppression by invertebrate predators: a meta‐analysis
Source: Ecology. 2018 Jul 5;99(8):1771–82. doi: 10.1002/ecy.2378 (PMC6099248; doi:10.1002/ecy.2378)
Supplement: Supplementary file 8 [file ECY-99-1771-s008.docx]

**Appendix S8**

As study design had a significant effect on the SMD_max_ metric _(_predator polyculture compared to the most effective predator species in a monoculture) (see Main paper; Table 3). We re-analysed SMD_max_ removing additive design studies that did not account for predator density. This left 140 data points from a total of 26 studies. The model results from this subset of data supported our main result that functional diversity had a positive effect on SMD_max_ (Table 1 and 2). The only difference was the absence of ratio_small_ from the 2AIC_c_ subset. However, our main analysis showed large variation of the impact of ratio_small_ on SMD_max_. Thus, indicating no clear positive or negative effect of this variable.

**Table S1**. 2AIC_c_ model subset for SMD_max ._

| **Rank** | **Model** | **AIC_c_** | **Weight** | | **Relative weight** | |
| --- | --- | --- | --- | --- | --- | --- |
| 1 | Functional diversity + Predator richness | 355.877 | | 0.122 | | 0.385 |
| 2 | Functional diversity + Predator richness + Phylogenetic diversity | 356.524 | | 0.089 | | 0.279 |
| 3 | Functional diversity + Predator richness + Prey size | 357.294 | | 0.060 | | 0.190 |
| 4 | Functional diversity + Predator richness + Size difference | 357.794 | | 0.047 | | 0.148 |

| **Parameter** | **Estimate** | **Importance** | **95% CI lower bound** | **95% CI upper bound** |
| --- | --- | --- | --- | --- |
| Size difference | 0.002 | 0.148 | -0.01 | 0.014 |
| Prey size | -0.023 | 0.189 | -0.122 | 0.076 |
| Phylogenetic diversity | 0.161 | 0.278 | -0.412 | 0.734 |
| **Predator richness >2** | -0.487 | 1.000 | -0.794 | -0.18 |
| **Functional diversity** | 0.688 | 1.000 | 0.067 | 1.309 |

**Table S2.** Multimodel average parameter estimates for SMD_max._ Predator richness estimate is the difference between the reference level (predator richness = 2 species). Parameters in bold indicate that the variable was included in the highest ranked model.
